# Supplementary material for: Chronic kidney disease biomarkers and mortality among older adults: A comparison study of survey samples in China and the United States
Source: PLoS One. 2022 Jan 12;17(1):e0260074. doi: 10.1371/journal.pone.0260074 (PMC8754291; doi:10.1371/journal.pone.0260074)
Supplement: S1 Table — (PDF) [file pone.0260074.s001.pdf]

S1 Table. Cohorts' characteristics of this study.

COUNRTRY	China	The US
Cohort name	The Chinese Longitudinal Healthy Longevity Survey Biomarkers Cohort (Healthy Ageing and Biomarkers Cohort Study (HABCS))	National Health and Nutrition Examination Survey (NHANES)
Study year	2012-2018	2011-2014, followed up in 2015
Study participants	Older men and women aged 65 or above were included in this study	
Sample methods	As far as possible, for each centenarian interviewee, one nearby octogenarian, one nearby nonagenarian and one older adult aged 70–79 years old of predefined age and sex are randomly interviewed. In addition, 0.5 nearby elderly aged 65–69 of predefined age and sex are also randomly interviewed.	Two sample persons per eligible household;<br>Oversampled: Hispanic persons, non-Hispanic black persons, non-Hispanic non-black Asian persons, low-income non-Hispanic non-black non-Asian white and other persons (at or below 130% of federal poverty level), and adults aged 80 and over
Sample size	2019	2177
Study areas	Eight longevity areas in China, which cover the northern, middle and southern parts of China	The 50 states of the US and District of Columbia
Biomarker measurement	The urine was tested for microalbumin and creatinine using Siemens Microalbustix (Siemen Healthcare Diagnostic, USA). Blood plasma analyses were determined by an Automatic Biochemistry Analyzer (Hitachi 7180, Japan) using commercially available diagnostic kits (Roche Diagnostic, Mannheim, Germany), and serum creatinine was determined by the picric acid method, BUN was determined by urease ultraviolet rate method, and blood uric acid was determined by uricase colorimetric method. The central clinical laboratory at Capital Medical University conducted all laboratory analyses in Beijing.	Urinary albumin was measured by a solid-phase fluorescent Immunoassay described by Chavers et al. (14). In 2011-2012, urinary creatinine was measured on the Roche/Hitachi Mod P chemistry analyzer; while in 2013-2014, it was measured on the Roche/Hitachi Cobas 6000 chemistry analyzer. Urine specimens were analyzed in the University of Minnesota, Minneapolis, MN. DxC800 determined the concentration of serum creatinine, BUN, plasma albumin and uric acid by means of the Jaffe rate method, the enzymatic conductivity rate method, a bichromatic digital endpoint method and a timed endpoint method, respectively. Serum specimens were shipped to the Collaborative Laboratory Services, Ottumwa, Iowa for analysis.
Mortality data	The immediate family members of subjects reported the mortality information in the follow-up surveys in 2014 and 2017/2018.	Link to the National Death Index (NDI)
Covariates	Age, sex, race/ethnicities, education, household income, marital status, health condition, smoking status, drinking status, physical activity, and body mass index (BMI), hypertension and diabetes	Age, sex, race/ethnicities, education, household income, marital status, health condition, smoking status, drinking status, physical activity, and body mass index (BMI), hypertension and diabetes
