# Supplementary material for: Chronic kidney disease biomarkers and mortality among older adults: A comparison study of survey samples in China and the United States
Source: PLoS One. 2022 Jan 12;17(1):e0260074. doi: 10.1371/journal.pone.0260074 (PMC8754291; doi:10.1371/journal.pone.0260074)
Supplement: S2 Table — 1. Characteristics of the included and excluded participants in CLHLS. 2. Characteristics of the included and excluded participants in NHANES. (ZIP) [file pone.0260074.s002.zip › S2-1 Table.pdf]

S2-1 Table. Characteristics of the included and excluded participants in CLHLS.

Characteristics	All participants in 2011-2012 (n=2354)	Participants with missing biomarkers (n=335)	Participants without missing biomarkers (n=2019)		
			Overall	Lost in first follow-up (n=321)	Not lost in first follow-up (n=1698)
Age (mean ± SD)	86.5 (12.2)	91.7 (11.2)	85.7±12.2	85.1±12.4	85.8±12.2
**Age group**					
65-69	255 (10.8)	15 (4.5)	240 (11.9)	45 (14.0)	195 (11.5)
70-74	260 (11.0)	20 (6.0)	240 (11.9)	40 (12.5)	200 (11.8)
75-79	234 (9.9)	17 (5.1)	217 (10.7)	36 (11.2)	181 (10.7)
80+	1605 (68.2)	283 (84.5)	1322 (65.5)	200 (62.3)	1122 (66.1)
**Gender**					
Male	1031 (43.8)	98 (29.3)	933 (46.2)	156 (48.6)	777 (45.8)
Female	1323 (56.2)	237 (70.7)	1086 (53.8)	165 (51.4)	921 (54.2)
**Race**					
Han Chinese	2124 (90.2)	307 (91.6)	1817 (90.0)	290 (90.3)	1527 (89.9)
Ethnic minorities	175 (7.4)	23 (6.9)	152 (7.5)	21 (6.5)	131 (7.7)
Missing	55 (2.3)	5 (1.5)	50 (2.5)	10 (3.1)	40 (2.4)
**Education**					
No formal education	1481 (62.9)	243 (72.5)	1238 (61.3)	178 (55.5)	1060 (62.4)
Formal education	850 (36.1)	86 (25.7)	764 (37.8)	137 (42.7)	627 (36.9)
Missing	23 (1.0)	6 (1.8)	17 (0.8)	6 (1.9)	11 (0.6)
**Household income (RMB)**					
Tertile 1 (<6,000)	741 (31.5)	104 (31.0)	637 (31.6)	80 (24.9)	557 (32.8)
Tertile 2 (6000-19,000)	750 (31.9)	89 (26.6)	661 (32.7)	84 (26.2)	577 (34.0)
Tertile 3 (20,000-over 100,000)	674 (28.6)	102 (30.4)	572 (28.3)	120 (37.4)	452 (26.6)
Missing	189 (8.0)	40 (11.9)	149 (7.4)	37 (11.5)	112 (6.6)
**Marital Status**					
Married	840 (35.7)	66 (19.7)	774 (38.3)	123 (38.3)	651 (38.3)
Separated	45 (1.9)	5 (1.5)	40 (2.0)	8 (2.5)	32 (1.9)
Divorced	5 (0.2)	0 (0)	5 (0.2)	1 (0.3)	4 (0.2)
Widowed	1380 (58.6)	249 (74.3)	1131 (56.0)	171 (53.3)	960 (56.5)
Never married	26 (1.1)	6 (1.8)	20 (1.0)	3 (0.9)	17 (1.0)
Missing	58 (2.5)	9 (2.7)	49 (2.4)	15 (4.7)	34 (2.0)
**Health condition**					
Very good	115 (4.9)	12 (3.6)	103 (5.1)	20 (6.2)	83 (4.9)
Good	821 (34.9)	71 (21.2)	750 (37.1)	108 (33.6)	642 (37.8)
Fair	909 (38.6)	134 (40.0)	775 (38.4)	119 (37.1)	656 (38.6)
Bad	257 (10.9)	56 (16.7)	201 (10.0)	41 (12.8)	160 (9.4)
Very Bad	18 (0.8)	6 (1.8)	12 (0.6)	2 (0.6)	10 (0.6)
Missing	234 (9.9)	56 (16.7)	178 (8.8)	31 (9.7)	147 (8.7)
**Smoking status**					
Never smoker	1713 (72.8)	248 (74.0)	1465 (72.6)	224 (69.8)	1241 (73.1)
Former smoker	191 (8.1)	27 (8.1)	164 (8.1)	25 (7.8)	139 (8.2)
Current smoker	383 (16.3)	49 (14.6)	334 (16.5)	57 (17.8)	277 (16.3)

Missing	67 (2.8)	11 (3.3)	56 (2.8)	15 (4.7)	41 (2.4)
**Drinking status**					
Never drinker	1807 (76.8)	279 (83.3)	1528 (75.7)	243 (75.7)	1285 (75.7)
Former drinker	138 (5.9)	18 (5.4)	120 (5.9)	18 (5.6)	102 (6.0)
Current drinker	342 (14.5)	27 (8.1)	315 (15.6)	44 (13.7)	271 (16.0)
Missing	67 (2.8)	11 (3.3)	56 (2.8)	16 (5.0)	40 (2.4)
**Physical activity**					
Yes	345 (14.7)	34 (10.1)	311 (15.4)	244 (14.4)	67 (20.9)
No	1884 (80.0)	286 (85.4)	1598 (79.1)	1373 (80.9)	225 (70.1)
Missing	125 (5.3)	15 (4.5)	110 (5.4)	81 (4.8)	29 (9.0)
**Body mass index (kg/m^2)**					
Underweight (<18.5)	575 (24.4)	98 (29.3)	477 (23.6)	83 (25.9)	394 (23.2)
Normal (18.5-24.9)	1316 (55.9)	163 (48.7)	1153 (57.1)	175 (54.5)	978 (57.6)
Overweight (25.0-29.9)	239 (10.2)	10 (3.0)	229 (11.3)	31 (9.7)	198 (11.7)
Obese (>=30)	65 (2.8)	7 (2.1)	58 (2.9)	11 (3.4)	47 (2.8)
Missing	159 (6.8)	57 (17.0)	102 (5.1)	21 (6.5)	81 (4.8)

Data are mean (SD) and n (%).
Abbreviations: SD = standard deviation, RMB = renminbi.
