# Supplementary material for: Chronic kidney disease biomarkers and mortality among older adults: A comparison study of survey samples in China and the United States
Source: PLoS One. 2022 Jan 12;17(1):e0260074. doi: 10.1371/journal.pone.0260074 (PMC8754291; doi:10.1371/journal.pone.0260074)
Supplement: S2 Table — 1. Characteristics of the included and excluded participants in CLHLS. 2. Characteristics of the included and excluded participants in NHANES. (ZIP) [file pone.0260074.s002.zip › S2-2 Table.pdf]

S2-2 Table. Characteristics of the included and excluded participants in NHANES.

Characteristics	All participants in 2011-2014 (n=2556)	Participants with missing biomarkers (n=379)
**Age (mean ± SD)**	73.5±5.4	74.7±5.4
**Age group**		
65-69	772 (30.2)	90 (23.8)
70-74	645 (25.2)	78 (20.6)
75-79	424 (16.6)	63 (16.6)
80+	715 (28.0)	148 (39.1)
**Gender**		
Male	1226 (48.0)	154 (40.6)
Female	1330 (52.0)	225 (59.4)
**Race/Ethnicity**		
Mexican American	192 (7.5)	23 (6.1)
Other Hispanics	230 (9.0)	42 (11.1)
Non-Hispanic White	1314 (51.4)	163 (43.0)
Non-Hispanic Black	546 (21.4)	107 (28.2)
Non-Hispanic Asian	230 (9.0)	34 (9.0)
Other races	44 (1.7)	10 (2.6)
**Education**		
Below high school	792 (31.0)	143 (37.7)
High school	586 (22.9)	82 (21.6)
College or above	1170 (45.8)	151 (39.8)
Missing	8 (0.3)	3 (0.8)
**Income (PIR)**		
Tertile 1 (0-1.87)	1116 (43.7)	188 (49.6)
Tertile 2 (1.88-3.86)	657 (25.7)	75 (19.8)
Tertile (>=3.87)	537 (21.0)	63 (16.6)
Missing	246 (9.6)	53 (14.0)
**Marital Status**		
Married	1337 (52.3)	164 (43.3)
Separated	53 (2.1)	9 (2.4)
Divorced	296 (11.6)	38 (10.0)
Widowed	706 (27.6)	144 (38.0)
Never married	114 (4.5)	14 (3.7)
Living with partner	46 (1.8)	7 (1.9)
Missing	4 (0.2)	3 (0.8)
**Health condition**		
Excellent	174 (6.8)	16 (4.2)
Very good	541 (21.2)	26 (6.9)
Good	878 (34.4)	67 (17.7)
Fair	578 (22.6)	69 (18.2)
Poor	118 (4.6)	23 (6.1)
Missing	267 (10.5)	178 (47.0)

**Smoking status**		
Never smoker	1282 (50.2)	186 (49.1)
Former smoker	1015 (39.7)	158 (41.7)
Current smoker	255 (10.0)	33 (8.7)
Missing	4 (0.2)	2 (0.5)
**Drinking status**		
Never drinker	442 (17.3)	50 (13.2)
Former drinker	361 (14.1)	43 (11.4)
Current drinker	1458 (57.0)	102 (26.9)
Missing	295 (11.5)	184 (48.6)
**Physical activity**		
Yes	976 (38.2)	108 (28.5)
No	1576 (61.7)	270 (71.2)
Missing	4 (0.2)	1 (0.3)
**Body mass index (kg/m^2)**		
Underweight (<18.5)	44 (1.7)	8 (2.1)
Normal (18.5-24.9)	658 (25.7)	79 (20.8)
Overweight (25.0-29.9)	840 (32.9)	64 (16.9)
Obese (>=30)	826 (32.3)	80 (21.1)
Missing	188 (7.4)	148 (39.1)
**Hypertension**		
Yes	846 (33.1)	100 (26.4)
No	1710 (66.9)	279 (73.6)
**Diabetes**		
Yes	629 (24.6)	103 (27.2)
No	1925 (75.3)	275 (72.6)
Missing	2 (0.1)	1 (0.2)

Data are mean (SD) and n (%).
Abbreviations: SD = standard deviation, PIR = ratio of family income to poverty.
