# Supplementary material for: Chronic kidney disease biomarkers and mortality among older adults: A comparison study of survey samples in China and the United States
Source: PLoS One. 2022 Jan 12;17(1):e0260074. doi: 10.1371/journal.pone.0260074 (PMC8754291; doi:10.1371/journal.pone.0260074)
Supplement: S3 Table — (PDF) [file pone.0260074.s003.pdf]

S3 Table. Equations used to estimate glomerular filtration rate eGFR (CKD-EPI: Chronic Kidney Disease Epidemiology Collaboration) [1].

Race	Gender	Serum creatinine (Cr, unit: mg/dL)	Equation (eGFR, unit: mL/min per 1.73 m^2)
White or other	Female	≤ 0.7	$144 \times (\frac{Cr}{0.7})^{-0.329} \times 0.993^{age}$
White or other	Female	> 0.7	$144 \times (\frac{Cr}{0.7})^{-1.209} \times 0.993^{age}$
White or other	Male	≤ 0.9	$141 \times (\frac{Cr}{0.9})^{-0.411} \times 0.993^{age}$
White or other	Male	> 0.9	$141 \times (\frac{Cr}{0.9})^{-1.209} \times 0.993^{age}$
Black	Female	≤ 0.7	$166 \times (\frac{Cr}{0.7})^{-0.329} \times 0.993^{age}$
Black	Female	> 0.7	$166 \times (\frac{Cr}{0.7})^{-1.209} \times 0.993^{age}$
Black	Male	≤ 0.9	$163 \times (\frac{Cr}{0.9})^{-0.411} \times 0.993^{age}$
Black	Male	> 0.9	$163 \times (\frac{Cr}{0.9})^{-1.209} \times 0.993^{age}$

References

1. Levey AS, Stevens LA, Schmid CH, Zhang YL, Castro AF, Feldman HI, et al. A new equation to estimate glomerular filtration rate. Annals of internal medicine. 2009;150(9):604-12.
