# Supplementary material for: Chronic kidney disease biomarkers and mortality among older adults: A comparison study of survey samples in China and the United States
Source: PLoS One. 2022 Jan 12;17(1):e0260074. doi: 10.1371/journal.pone.0260074 (PMC8754291; doi:10.1371/journal.pone.0260074)
Supplement: S4 Table — 1. Demographic characteristics and median (P25-P75) of biomarkers (Chinese participants: CLHLS 2012). 2. Demographic characteristics and median (P25-P75) of biomarkers (US participants: NHANES 2011–2014). (ZIP) [file pone.0260074.s004.zip › S4-1 Table.pdf]

S4-1 Table. Demographic characteristics and median (P25-P75) of biomarkers (Chinese participants: CLHLS 2012)

Characteristics	n (%)	Urine microalbumin (mg/L)	Urinary creatinine (mg/dL)	Albumin creatinine ratio (mg/g)	Serum creatinine (μmol/L)	Blood urea nitrogen (mmol/L)	Plasma albumin (g/L)	Uric acid (μmol/L)	eGFR (mL/min per 1.73 m2)
**Total**	2019 (100)	5 (1, 18.8)	93.2 (57.3, 141.1)	5.8 (1.2, 21.9)	77 (65, 92)	6.6 (5.5, 7.9)	40.4 (37.1, 43.6)	281.2 (230.9, 343.3)	69.6 (55.1, 81.2)
**Range [min, max]**	/								
**Age (mean ± SD)**	85.7±12.2	/	/	/	/	/	/	/	/
**Age group**									
65-69	240 (11.9)	1.6 (0,8.3)	113.9 (77.8,160.8)	1.3 (0,6.6)	72.5 (61.8,87)	6.1 (5.2,7.3)	42.1 (39.6,44.8)	266.6 (220.4,336)	87.7 (76.1,94.2)
70-74	240 (11.9)	2.7 (0.5,10.2)	100.3 (68.8,141.2)	3 (0.7,11.2)	75 (65,88)	6.2 (5,7.4)	42.4 (39.7,45.5)	284.8 (231.8,336.4)	82.3 (71.3,89.1)
75-79	217 (10.7)	3.8 (0.7,13.6)	110 (70.7,149.3)	3.5 (0.8,12.1)	78 (67,94)	6.3 (5.2,7.6)	41 (38.5,44.1)	284 (233.3,342)	78 (61.7,85.3)
80+	1322 (65.5)	6.8 (1.6,24.1)	84.8 (49.1,134.1)	8.3 (2.3,30.2)	78 (65,96)	6.7 (5.6,8.2)	39.4 (36.1,42.7)	282.9 (233.4,345.5)	62.8 (50.2,74.5)
**Gender**									
Male	933 (46.2)	3.5 (0.5,14.2)	113.7 (74.5,160.8)	3.4 (0.6,12.3)	84 (72,98)	6.6 (5.6,7.9)	40.8 (37.9,43.9)	303.8 (252.3,362.5)	73.8 (60.4,85.3)
Female	1086 (53.8)	6.7 (1.6,23.1)	76.7 (46.3,119.1)	8.8 (2.5,31.4)	71 (60,85)	6.5 (5.4,7.9)	40 (36.7,43.4)	265.2 (212.4,321.8)	65.8 (51.3,77)
**Race**									
Han Chinese	1817 (90.0)	4.8 (1,17.5)	91.8 (55.3,137.8)	5.6 (1.2,22.3)	77 (64,92)	6.6 (5.5,7.9)	40.6 (37.4,43.7)	280.1 (230.7,341.4)	69.7 (55.5,81.6)
Ethnic minorities	152 (7.5)	6.9 (1.2,24.3)	124.1 (82.4,180.2)	6.9 (1.3,16.2)	83 (69.8,96.3)	6.5 (5.6,8)	38.2 (35.4,41.2)	307.1 (244.5,366)	65.4 (50.7,78)
Missing	50 (2.5)	12.9 (1.3,34.2)	87.8 (62.5,109.5)	10.1 (2,31.2)	77 (61,89.8)	6.6 (5.8,7.6)	39.4 (37.3,42.5)	262 (209.1,325.4)	73 (48.8,78.1)
**Education**									
No formal education	1238 (61.3)	6.5 (1.5,23.2)	83.3 (49.1,128.6)	8.3 (2.2,29)	74 (62,89)	6.6 (5.5,7.9)	39.8 (36.7,42.8)	272.3 (221,331.1)	66.4 (52.5,77.6)
Formal education	764 (37.8)	3.2 (0.5,12.7)	108.4 (71.8,156.3)	3 (0.5,11.7)	82 (68.8,96)	6.5 (5.4,7.8)	41.5 (38.2,44.5)	299.8 (246.9,359.4)	75.1 (60.3,86.2)
Missing	17 (0.8)	13.1 (4.4,30.9)	89.8 (45.6,138.8)	18.6 (5.2,46.7)	79 (67,99)	6.6 (5.8,7.9)	38.2 (35.2,41.7)	273.4 (239.8,342.9)	66.7 (46.9,77.1)
**Household income (RMB)**									
Tertile 1 (<6,000)	637 (31.6)	4.4 (0.9,16.6)	92.5 (60.1,136.7)	4.9 (1.1,18.6)	73 (62,88)	6.6 (5.6,7.8)	40 (37.1,43)	263.9 (211.7,320)	73.2 (60.2,83.2)
Tertile 2 (6,000-19,000)	661 (32.7)	4.7 (0.9,17.1)	94.4 (59.8,146.6)	5.3 (1,21.2)	77 (65,92)	6.6 (5.5,7.9)	40.4 (37.3,43.7)	288.3 (235.9,343.3)	69.2 (55.7,80.5)
Tertile 3 (20,000-more than 100,000)	572 (28.3)	5.2 (1.3,21.5)	94 (53.8,143.2)	7.3 (1.5,25.6)	81 (68,99)	6.4 (5.3,7.9)	40.8 (37.4,44.4)	300.6 (250.5,358.6)	65.6 (51,79.2)
Missing	149 (7.4)	8.1 (1.4,24)	81.4 (54.6,134.8)	10.7 (2.8,29.8)	78 (62,98)	6.5 (5.3,8)	40.3 (35.6,43.6)	287 (228.1,366.7)	66.4 (49,78)
**Marital Status**									
Married	774 (38.3)	2.8 (0.5,12.1)	108.5 (70.7,155.9)	2.9 (0.5,11.9)	79 (66,93)	6.4 (5.3,7.7)	41.6 (38.7,44.8)	288.3 (239.2,348.6)	77.4 (64.3,87.4)

Separated	40 (2.0)	3.4 (0.7,10.1)	86.6 (58.4,159.1)	4.1 (0.7,15.5)	87.5 (68.8,98.8)	6.1 (5.2,7.4)	39.4 (35.5,41.7)	314.4 (252.5,347.4)	71.1 (56.2,81.4)
Divorced	5 (0.2)	3.1 (0.3,6.2)	67.1 (66,127.4)	1.9 (0.4,7.1)	64 (57,77)	7.5 (6.4,7.6)	40.7 (37.4,40.8)	235 (209.6,320.1)	90.5 (83.1,100.3)
Widowed	1131 (56.0)	6.8 (1.7,23.6)	82.1 (47.7,128.6)	8.4 (2.5,30.8)	76 (63.5,91.5)	6.7 (5.5,8.1)	39.4 (36.3,42.6)	275.2 (224.8,336.4)	63.2 (50.7,75.4)
Never married	20 (1.0)	2.1 (0.1,10.4)	109.3 (79.6,132.6)	1.6 (0.1,12.1)	79.5 (66.8,94.5)	6.3 (5.7,7.6)	41.4 (37.4,43.6)	260.4 (226.5,343.9)	81.2 (60.3,91.9)
Missing	49 (2.4)	10.3 (2.2,27.1)	88.5 (55.1,143.4)	11.3 (3.5,27.8)	76 (65,92)	6.3 (5.3,7.5)	41.5 (38.3,43.9)	327.6 (263.7,401)	66.9 (53.2,77.3)
**Health condition**									
Very good	103 (5.1)	3.9 (0.9,13.7)	99.2 (64.4,156.4)	4.1 (1.2,12.8)	80 (69,92)	6.6 (5.3,7.5)	41 (37.5,44)	299.8 (237.1,361.1)	69.2 (54.6,81.7)
Good	750 (37.1)	4.1 (0.9,16.7)	94.6 (59.7,141.2)	4.9 (1,15.5)	76 (65,91)	6.6 (5.6,8)	40.5 (37.4,43.8)	279.4 (229.1,343.4)	72.3 (57.1,83.2)
Fair	775 (38.4)	5.6 (1.1,19.6)	93.8 (54.2,141.4)	6.4 (1.5,25.6)	78 (65,94)	6.6 (5.5,7.8)	40.7 (37.9,43.9)	281.7 (234.9,339.5)	67.8 (54.3,80.2)
Bad	201 (10.0)	6 (1,17.7)	89.6 (59.2,133.5)	7.2 (1,22.3)	79 (66,97)	6.3 (5.1,7.6)	39.4 (36.2,43)	287.7 (239.5,341.4)	67.8 (51.5,81.2)
Very Bad	12 (0.6)	5.5 (2.7,18.1)	66.1 (46.9,104.8)	5.5 (3.3,27.6)	70 (56.3,76.8)	6.1 (5,7)	40.1 (31.3,42)	241.5 (211.3,286.6)	77.4 (65.3,83.7)
Missing	178 (8.8)	7.8 (2.2,25)	84.1 (46.2,131.7)	10.5 (3.5,30.5)	73 (60,86.8)	6.7 (5.5,8)	38.4 (35.1,42.1)	272.9 (213.6,335.2)	66 (54.6,75.7)
**Smoking status**									
Never smoker	1465 (72.6)	5.2 (1.2,19.9)	88 (52.1,135.4)	6.6 (1.5,24.8)	75 (63,91)	6.6 (5.5,7.9)	40.3 (37.1,43.7)	275.1 (225.1,338.6)	68.2 (53.9,80.1)
Former smoker	164 (8.1)	4.3 (0.9,16)	105.2 (69.1,149)	4.2 (0.9,19.8)	85.5 (69.8,94.3)	6.7 (5.6,8.2)	40 (37,42.6)	296.6 (242.8,356.2)	69.4 (59.3,80.3)
Current smoker	334 (16.5)	3.8 (0.7,11.7)	116.2 (75.8,156.8)	3.7 (0.8,12)	82 (71,96)	6.4 (5.1,7.6)	40.6 (37.3,43.5)	301 (246.9,350.7)	76.2 (61.3,86.5)
Missing	56 (2.8)	9.4 (2,31.1)	87.6 (55,131.1)	11.7 (2.6,33)	77 (65.8,89.5)	6.4 (5.3,7.8)	41.8 (40.2,44.3)	320.4 (254.9,396)	67.1 (55.6,78.5)
**Drinking status**									
Never drinker	1528 (75.7)	5.3 (1.2,19.2)	90.4 (54.5,137.6)	6.5 (1.5,23.2)	76 (63,92)	6.6 (5.5,7.9)	40.2 (37.1,43.5)	275.1 (225.1,336.5)	68.5 (54,80.3)
Former drinker	120 (5.9)	4.7 (0.8,21.8)	105.2 (57.9,150.9)	4.2 (0.9,21.1)	85.5 (70.8,96)	6.6 (5.6,7.9)	39.6 (36.3,42.5)	312.3 (251.4,355)	68.1 (54.8,80.8)
Current drinker	315 (15.6)	3.5 (0.6,13.3)	106.9 (68.4,147.9)	3.6 (0.7,14.6)	79 (69,93)	6.4 (5.1,7.6)	41 (38,44)	298.5 (245.7,363.9)	75.1 (61.7,85.2)
Missing	56 (2.8)	8.5 (2,25.8)	95.3 (60.5,148.4)	9.2 (1.6,26.3)	78.5 (65.8,92.3)	6.4 (5.3,7.9)	41.3 (38.2,43.8)	330.9 (271.4,399.8)	66.7 (52.5,78.1)
**Physical activity**									
Yes	311 (15.4)	4.3 (0.8,16.6)	101.3 (64.2,163.2)	4.6 (0.9,17.8)	83 (69,98)	6.6 (5.5,7.9)	41.2 (38.2,44.4)	302.1 (247.2,359.9)	68.1 (55.7,81.3)
No	1598 (79.1)	4.9 (1,18.6)	91 (55.2,137.2)	5.9 (1.3,22.4)	76 (64,91)	6.6 (5.5,7.9)	40.2 (37,43.4)	277.3 (227.2,338)	69.7 (54.9,81.2)
Missing	110 (5.4)	9.9 (2.4,25.2)	104.5 (61.4,151.7)	9.6 (3.1,26.4)	75.5 (61,92)	6.1 (5.1,7.8)	41 (37.1,43.9)	311.8 (241.7,385.8)	72 (56.3,80.2)
**Body mass index (kg/m^2)**									
Underweight (<18.5)	477 (23.6)	6.3 (1.6,23.4)	85.5 (51.7,135.9)	8.3 (2.1,31.2)	78 (62,98)	6.6 (5.4,7.9)	39.6 (36.1,42.7)	276.9 (224.6,339.2)	63 (49,75.7)
Normal (18.5-24.9)	1153 (57.1)	4.3 (0.8,17)	95.3 (57.7,141.6)	4.9 (1,19.9)	77 (66,92)	6.6 (5.5,7.9)	40.5 (37.5,43.6)	278.8 (227.9,338.2)	71.4 (56.7,82.4)
Overweight (25.0-29.9)	229 (11.3)	5 (0.9,17.1)	106.9 (70.9,152.3)	4.5 (1,14)	78 (65,88)	6.6 (5.6,7.9)	42.1 (38.8,45.4)	306.4 (247.8,363.4)	75.2 (61.2,86.1)

Obese (>=30)	58 (2.9)	7.2 (2.5,23.6)	91.5 (57.7,137.3)	11.1 (2.4,22.2)	78.5 (63.3,91)	6.5 (5.2,8)	42.5 (38.2,45)	287.6 (247.4,356.5)	72 (55.8,82.1)
Missing	102 (5.1)	7.1 (1.5,20.2)	80.7 (40.1,121.9)	7.4 (3.7,31.4)	74 (61,92.8)	6.4 (5.2,7.5)	38.3 (35.3,41.5)	293.5 (250,344)	63.3 (52.3,72.9)
**Hypertension**									
Yes	1142 (56.6)	6.1 (1.3, 23.4)	84.7 (52.1, 133.3)	7.8 (1.8, 28.5)	78 (65, 92.8)	6.5 (5.5, 7.8)	40.7 (37.8, 43.8)	286.3 (236, 347.5)	68.1 (53.7, 80)
No	857 (42.4)	3.8 (0.7, 14.1)	103.3 (63.2, 148.2)	4.2 (0.8, 14.3)	76 (65, 92)	6.6 (5.5, 8)	40.1 (36.6, 43.2)	274.9 (223.8, 337.1)	71.6 (56.7, 82.8)
Missing	20 (1.0)	10 (3, 33.3)	119.8 (87.5, 186.4)	6.9 (4.4, 11.5)	86 (64.3, 110.8)	6.9 (6, 8)	35.4 (33.4, 38)	279.6 (234.5, 374.1)	63.7 (49, 77.1)
**Diabetes**									
Yes	48 (2.4)	5.4 (1.3, 31.3)	105.7 (56, 149.7)	4.7 (1.8, 27.1)	75.5 (64, 89.5)	6.6 (5.5, 7.5)	41.6 (38.3, 44.7)	299.1 (249.2, 355.2)	74.9 (55.9, 84.6)
No	1940 (96.1)	4.9 (1, 18.1)	93.1 (57.3, 140.8)	5.8 (1.2, 21.6)	77 (65, 92)	6.6 (5.5, 7.9)	40.4 (37.2, 43.6)	281 (230, 343)	69.5 (55.2, 81.2)
Missing	31 (1.5)	12.5 (4.3, 46.8)	104.5 (60.5, 157.4)	14.9 (4.4, 64.5)	85 (65, 97)	6.9 (5.5, 9.2)	36.3 (34, 43.1)	282.9 (245.3, 330.2)	59.7 (47.4, 71.2)

Abbreviations: P25 = 25th percentile, P75 = 75th percentile, eGFR = estimated glomerular filtration rate, RMB = renminbi.
