# Supplementary material for: Chronic kidney disease biomarkers and mortality among older adults: A comparison study of survey samples in China and the United States
Source: PLoS One. 2022 Jan 12;17(1):e0260074. doi: 10.1371/journal.pone.0260074 (PMC8754291; doi:10.1371/journal.pone.0260074)
Supplement: S5 Table — (PDF) [file pone.0260074.s005.pdf]

S5 Table. Odds ratio (95% CI) of factors associated with abnormal eGFR (<60 ml/min/1.73m^2) in CLHLS and NHANES.

NHANES				CLHLS			
**Characteristics**	**n (%)**	**OR (95% CI) ***	**P value**	**Characteristics**	**n (%)**	**OR (95% CI) ***	**P value**
Total	2177 (100)			Total	2019 (100)		
**Age group**				**Age group**			
65-69	682 (31.3)	Ref	\	65-69	240 (11.9)	Ref	\
70-74	567 (26.1)	**1.62 (1.20, 2.19)**	**0.002**	70-74	240 (11.9)	1.36 (0.72, 2.62)	0.348
75-79	361 (16.6)	**2.89 (2.10, 3.96)**	**<0.001**	75-79	217 (10.7)	**3.61 (2.02, 6.70)**	**<0.001**
80+	567 (26.1)	**5.24 (3.86, 7.12)**	**<0.001**	80+	1322 (65.5)	**7.89 (4.72, 13.91)**	**<0.001**
**Gender**				**Gender**			
Male	1072 (49.2)	Ref	\	Male	933 (46.2)	Ref	\
Female	1105 (50.8)	0.94 (0.75, 1.18)	0.586	Female	1086 (53.8)	**1.37 (1.05, 1.79)**	**0.022**
**Race/Ethnicity**				**Race**			
Mexican American	169 (7.8)	Ref	\	Han Chinese	1817 (90.0)	Ref	\
Other Hispanics	188 (8.6)	1.23 (0.71, 2.12)	0.466	Ethnic minorities	152 (7.5)	**1.66 (1.14, 2.43)**	**0.009**
Non-Hispanic White	1151 (52.9)	**1.98 (1.25, 3.12)**	**0.004**	Missing	50 (2.5)	1.05 (0.55, 1.97)	0.878
Non-Hispanic Black	439 (20.2)	**1.71 (1.07, 2.75)**	**0.026**				
Non-Hispanic Asian	196 (9.0)	1.08 (0.60, 1.92)	0.808				
Other races	34 (1.6)	**4.07 (1.75, 9.49)**	**0.001**				
**Education**				**Education**			
Below high school	649 (29.8)	Ref	\	No formal education	1238 (61.3)	Ref	\
High school	504 (23.2)	**0.75 (0.56, 0.99)**	**0.043**	Formal education	764 (37.8)	1.27 (0.96, 1.68)	0.098
College or above	1019 (46.8)	0.80 (0.61, 1.04)	0.094	Missing	17 (0.8)	0.95 (0.32, 2.74)	0.924
Missing	5 (0.2)	0.79 (0.13, 5.03)	0.807				
**Income (PIR)**				**Household income (RMB)**			
Tertile 1 (0-1.87)	928 (42.6)	Ref	\	Tertile 1 (<6,000)	637 (31.6)	Ref	\
Tertile 2 (1.88-3.86)	582 (26.7)	1.25 (0.97, 1.61)	0.089	Tertile 2 (6,000-19,000)	661 (32.7)	**1.56 (1.20, 2.03)**	**0.001**
Tertile (>=3.87)	474 (21.8)	1.25 (0.92, 1.68)	0.151	Tertile 3 (20,000-over 100,000)	572 (28.3)	**2.10 (1.61, 2.76)**	**<0.001**
Missing	193 (8.9)	0.81 (0.55, 1.19)	0.273	Missing	149 (7.4)	**1.69 (1.07, 2.64)**	**0.022**
**Marital Status**				**Marital Status**			
Married	1173 (53.9)	Ref	\	Married	774 (38.3)	Ref	\
Not married	1003 (46.1)	1.15 (0.93, 1.43)	0.207	Not married	1196 (59.2)	**1.38 (1.06, 1.80)**	**0.019**
Missing	1 (0.1)	NA	0.987	Missing	49 (2.4)	1.05 (0.28, 3.54)	0.945
**Health condition**				**Health condition**			
Excellent	158 (7.3)	Ref	\	Very good	103 (5.1)	Ref	\
Very good	515 (23.7)	0.75 (0.49, 1.16)	0.200	Good	750 (37.1)	0.86 (0.53, 1.44)	0.563
Good	811 (37.3)	1.04 (0.69, 1.57)	0.866	Fair	775 (38.4)	1.08 (0.66, 1.79)	0.768
Fair/Poor	604 (27.7)	1.25 (0.81, 1.93)	0.319	Bad/Very bad	213 (10.6)	1.25 (0.72, 2.20)	0.435
Missing	89 (4.1)	1.93 (0.63, 5.97)	0.251	Missing	178 (8.8)	0.74 (0.41, 1.35)	0.320
**Smoking status**				**Smoking status**			
Never smoker	1096 (50.3)	Ref	\	Never smoker	1465 (72.6)	Ref	\
Former smoker	857 (39.4)	1.10 (0.88, 1.39)	0.404	Former smoker	164 (8.1)	0.84 (0.55, 1.27)	0.419
Current smoker	222 (10.2)	1.39 (0.95, 2.02)	0.089	Current smoker	334 (16.5)	1.04 (0.73, 1.46)	0.838

Missing	2 (0.1)	1.50 (0.07, 32.62)	0.796	Missing	56 (2.8)	0.86 (0.25, 2.61)	0.803
**Drinking status**				**Drinking status**			
Never drinker	392 (18.0)	Ref	\	Never drinker	1528 (75.7)	Ref	\
Former drinker	318 (14.6)	1.13 (0.80, 1.60)	0.496	Former drinker	120 (5.9)	1.01 (0.64, 1.60)	0.956
Current drinker	1356 (62.3)	0.90 (0.66, 1.21)	0.466	Current drinker	315 (15.6)	0.75 (0.53, 1.04)	0.085
Missing	111 (5.1)	0.55 (0.21, 1.46)	0.232	Missing	56 (2.8)	2.41 (0.80, 7.15)	0.112
**Physical activity**				**Physical activity**			
Yes	868 (39.9)	Ref	\	Yes	311 (15.4)	Ref	\
No	1306 (60.0)	1.12 (0.90, 1.38)	0.305	No	1598 (79.1)	**0.69 (0.52, 0.93)**	**0.013**
Missing	3 (0.1)	NA	0.979	Missing	110 (5.4)	**0.51 (0.27, 0.94)**	**0.033**
**Body mass index (kg/m^2)**				**Body mass index (kg/m^2)**			
Underweight (<18.5)	36 (1.7)	Ref	\	Underweight (<18.5)	477 (23.6)	Ref	\
Normal (18.5-24.9)	579 (26.6)	2.12 (0.86, 5.23)	0.103	Normal (18.5-24.9)	1153 (57.1)	**0.73 (0.58, 0.94)**	**0.012**
Overweight (25.0-29.9)	776 (35.7)	2.37 (0.96, 5.84)	0.061	Overweight (25.0-29.9)	229 (11.3)	**0.60 (0.40, 0.90)**	**0.013**
Obese (>=30)	746 (34.3)	**2.80 (1.13, 6.94)**	**0.026**	Obese (>=30)	58 (2.9)	0.64 (0.33, 1.23)	0.188
Missing	40 (1.8)	2.96 (0.97, 9.06)	0.057	Missing	102 (5.1)	0.85 (0.54, 1.35)	0.503
**Hypertension**				**Hypertension**			
Yes	746 (34.3)	Ref	\	Yes	1142 (56.6)	Ref	\
No	1431 (65.7)	0.84 (0.68, 1.03)	0.095	No	857 (42.4)	0.89 (0.72, 1.10)	0.271
				Missing	20 (1.0)	1.26 (0.46, 3.39)	0.651
**Diabetes**				**Diabetes**			
Yes	526 (24.2)	Ref	\	Yes	48 (2.4)	Ref	\
No	1650 (75.8)	**0.50 (0.40, 0.64)**	**<0.001**	No	1940 (96.1)	0.86 (0.43, 1.82)	0.682
Missing	1 (0)	NA	0.989	Missing	31 (1.5)	1.09 (0.39, 3.12)	0.872

* The multi-variate analysis contained all the variables listed above in the logistic regression models.
