# Supplementary material for: Chronic kidney disease biomarkers and mortality among older adults: A comparison study of survey samples in China and the United States
Source: PLoS One. 2022 Jan 12;17(1):e0260074. doi: 10.1371/journal.pone.0260074 (PMC8754291; doi:10.1371/journal.pone.0260074)
Supplement: S6 Table — (PDF) [file pone.0260074.s006.pdf]

S6 Table. Odds ratio (95% CI) of factors associated with abnormal ACR (≥30 mg/g) in CLHLS and NHANES.

Characteristics	NHANES			CLHLS			
	n (%)	OR (95% CI) *	P value	Characteristics	n (%)	OR (95% CI) *	P value
Total	2177 (100)			Total	2019 (100)		
**Age group**				**Age group**			
65-69	682 (31.3)	Ref	\	65-69	240 (11.9)	Ref	\
70-74	567 (26.1)	1.22 (0.90, 1.67)	0.200	70-74	240 (11.9)	0.57 (0.30, 1.05)	0.076
75-79	361 (16.6)	**1.67 (1.19, 2.33)**	**0.003**	75-79	217 (10.7)	0.98 (0.55, 1.75)	0.950
80+	567 (26.1)	**2.56 (1.86, 3.53)**	**<0.001**	80+	1322 (65.5)	1.45 (0.92, 2.37)	0.122
**Gender**				**Gender**			
Male	1072 (49.2)	Ref	\	Male	933 (46.2)	Ref	\
Female	1105 (50.8)	0.80 (0.63, 1.02)	0.074	Female	1086 (53.8)	**1.51 (1.12, 2.06)**	**0.008**
**Race/Ethnicity**				**Race**			
Mexican American	169 (7.8)	Ref	\	Han Chinese	1817 (90.0)	Ref	\
Other Hispanics	188 (8.6)	0.90 (0.54, 1.51)	0.683	Ethnic minorities	152 (7.5)	0.86 (0.53, 1.34)	0.509
Non-Hispanic White	1151 (52.9)	0.86 (0.56, 1.33)	0.494	Missing	50 (2.5)	1.11 (0.54, 2.16)	0.762
Non-Hispanic Black	439 (20.2)	1.01 (0.65, 1.58)	0.956				
Non-Hispanic Asian	196 (9.0)	0.89 (0.51, 1.53)	0.662				
Other races	34 (1.6)	1.04 (0.42, 2.54)	0.938				
**Education**				**Education**			
Below high school	649 (29.8)	Ref	\	No formal education	1238 (61.3)	Ref	\
High school	504 (23.2)	1.03 (0.76, 1.38)	0.864	Formal education	764 (37.8)	0.77 (0.56, 1.05)	0.102
College or above	1019 (46.8)	0.98 (0.73, 1.30)	0.862	Missing	17 (0.8)	1.59 (0.52, 4.50)	0.392
Missing	5 (0.2)	2.62 (0.40, 17.29)	0.318				
**Income (PIR)**				**Household income (RMB)**			
Tertile 1 (0-1.87)	928 (42.6)	Ref	\	Tertile 1 (<6,000)	637 (31.6)	Ref	\
Tertile 2 (1.88-3.86)	582 (26.7)	0.93 (0.71, 1.23)	0.618	Tertile 2 (6,000-19,000)	661 (32.7)	1.05 (0.79, 1.41)	0.73
Tertile (>=3.87)	474 (21.8)	0.90 (0.64, 1.25)	0.513	Tertile 3 (20,000-over 100,000)	572 (28.3)	**1.50 (1.11, 2.02)**	**0.008**
Missing	193 (8.9)	0.95 (0.65, 1.40)	0.803	Missing	149 (7.4)	1.18 (0.71, 1.93)	0.51
**Marital Status**				**Marital Status**			
Married	1173 (53.9)	Ref	\	Married	774 (38.3)	Ref	\
Not married	1003 (46.1)	1.09 (0.86, 1.37)	0.497	Not married	1196 (59.2)	1.16 (0.86, 1.59)	0.338
Missing	1 (0.1)	NA	0.983	Missing	49 (2.4)	0.98 (0.20, 4.14)	0.973
**Health condition**				**Health condition**			
Excellent	158 (7.3)	Ref	\	Very good	103 (5.1)	Ref	\
Very good	515 (23.7)	0.90 (0.53, 1.52)	0.696	Good	750 (37.1)	1.18 (0.65, 2.24)	0.606
Good	811 (37.3)	1.24 (0.76, 2.04)	0.396	Fair	775 (38.4)	1.59 (0.89, 3.02)	0.133
Fair/Poor	604 (27.7)	**2.06 (1.24, 3.42)**	**0.006**	Bad/Very bad	213 (10.6)	1.30 (0.67, 2.63)	0.444
Missing	89 (4.1)	**3.62 (1.06, 12.40)**	**0.040**	Missing	178 (8.8)	1.46 (0.73, 3.02)	0.293
**Smoking status**				**Smoking status**			
Never smoker	1096 (50.3)	Ref	\	Never smoker	1465 (72.6)	Ref	\
Former smoker	857 (39.4)	1.16 (0.90, 1.49)	0.247	Former smoker	164 (8.1)	0.94 (0.57, 1.52)	0.816
Current smoker	222 (10.2)	1.45 (0.99, 2.14)	0.060	Current smoker	334 (16.5)	0.96 (0.64, 1.42)	0.835
Missing	2 (0.1)	1.70 (0.06, 45.06)	0.750	Missing	56 (2.8)	2.57 (0.82, 7.64)	0.093

**Drinking status**				**Drinking status**			
Never drinker	392 (18.0)	Ref	\	Never drinker	1528 (75.7)	Ref	\
Former drinker	318 (14.6)	1.17 (0.81, 1.68)	0.411	Former drinker	120 (5.9)	1.28 (0.75, 2.12)	0.344
Current drinker	1356 (62.3)	0.81 (0.59, 1.11)	0.190	Current drinker	315 (15.6)	1.03 (0.70, 1.50)	0.877
Missing	111 (5.1)	0.40 (0.14, 1.16)	0.092	Missing	56 (2.8)	0.40 (0.07, 1.73)	0.262
**Physical activity**				**Physical activity**			
Yes	868 (39.9)	Ref	\	Yes	311 (15.4)	Ref	\
No	1306 (60.0)	0.91 (0.72, 1.14)	0.389	No	1598 (79.1)	0.93 (0.66, 1.31)	0.652
Missing	3 (0.1)	2.38 (0.19, 29.96)	0.502	Missing	110 (5.4)	1.13 (0.57, 2.15)	0.726
**Body mass index (kg/m^2)**				**Body mass index (kg/m^2)**			
Underweight (<18.5)	36 (1.7)	Ref	\	Underweight (<18.5)	477 (23.6)	Ref	\
Normal (18.5-24.9)	579 (26.6)	0.64 (0.30, 1.39)	0.258	Normal (18.5-24.9)	1153 (57.1)	0.87 (0.66, 1.14)	0.294
Overweight (25.0-29.9)	776 (35.7)	0.59 (0.27, 1.27)	0.173	Overweight (25.0-29.9)	229 (11.3)	0.58 (0.36, 0.93)	0.025
Obese (>=30)	746 (34.3)	0.71 (0.33, 1.54)	0.385	Obese (>=30)	58 (2.9)	0.98 (0.47, 1.90)	0.948
Missing	40 (1.8)	2.23 (0.81, 6.13)	0.120	Missing	102 (5.1)	0.86 (0.51, 1.43)	0.565
**Hypertension**				**Hypertension**			
Yes	746 (34.3)	Ref	\	Yes	1142 (56.6)	Ref	\
No	1431 (65.7)	**0.49 (0.39, 0.61)**	**<0.001**	No	857 (42.4)	**0.56 (0.44, 0.72)**	**<0.001**
				Missing	20 (1.0)	0.77 (0.21, 2.29)	0.664
**Diabetes**				**Diabetes**			
Yes	526 (24.2)	Ref	\	Yes	48 (2.4)	Ref	\
No	1650 (75.8)	**0.40 (0.32, 0.51)**	**<0.001**	No	1940 (96.1)	0.62 (0.31, 1.34)	0.201
Missing	1 (0)	NA	0.983	Missing	31 (1.5)	0.92 (0.32, 2.68)	0.878

* The multi-variate analysis contained all the variables listed above in the logistic regression models.
