# Supplementary material for: Chronic kidney disease biomarkers and mortality among older adults: A comparison study of survey samples in China and the United States
Source: PLoS One. 2022 Jan 12;17(1):e0260074. doi: 10.1371/journal.pone.0260074 (PMC8754291; doi:10.1371/journal.pone.0260074)
Supplement: S7 Table — 1. Demographic characteristics and weighted mean (SD) of biomarkers (Chinese participants: CLHLS 2012). 2. Demographic characteristics and weighted mean (SD) of biomarkers (US participants: NHANES 2011–2014). (ZIP) [file pone.0260074.s007.zip › S7-1 Table.pdf]

S7-1 Table. Demographic characteristics and weighted mean (SD) of biomarkers (Chinese participants: CLHLS 2012).

Characteristics	n (%)	CKD n (%)	Urine microalbumin (mg/L)	Urinary creatinine (mg/dL)	Albumin creatinine ratio (mg/g)	Serum creatinine (μmol/L)	Blood urea nitrogen (mmol/L)	Plasma albumin (g/L)	Uric acid (μmol/L)	eGFR (mL/min per 1.73 m^2)
**Total**	1981 (100)	871 (28.5)	18.4 (61.6)	117.1 (69.3)	19.1 (74.3)	79.0 (24.0)	6.4 (1.8)	42.1 (4.4)	290.5 (91.1)	75.4 (16.4)
**Age group**										
65-69	240 (12.1)	43 (18.5)	15.4 (50.7)	126.4 (71.5)	16.6 (53.8)	75 (20.3)	6.3 (1.5)	42.9 (4)	282.5 (86.6)	82.5 (14.2)
70-74	240 (12.1)	44 (19.6)	10.1 (17.7)	110.6 (63.9)	11.3 (23.3)	76.9 (19.3)	6.3 (1.7)	42.4 (4.4)	285.6 (84.6)	78 (14.1)
75-79	217 (11.0)	69 (34.2)	25.6 (89.7)	118 (70.3)	20.3 (59)	83.2 (28.5)	6.5 (1.9)	41.5 (4.4)	294 (102.1)	71.1 (16.1)
80+	1284 (64.8)	715 (49.6)	26.5 (75.7)	109.1 (69.5)	31.5 (131.6)	84 (28.3)	6.9 (2.1)	41.2 (4.7)	305.6 (93)	65.1 (16.4)
**Gender**										
Male	928 (46.8)	304 (24.2)	20.8 (77.6)	131.7 (73.7)	18 (64.7)	87.2 (25.3)	6.5 (1.7)	42 (4.6)	317.1 (88.9)	76.6 (16.3)
Female	1053 (53.2)	567 (32.6)	16.2 (40.2)	102.9 (61.5)	20.2 (82.5)	71.1 (19.7)	6.4 (1.8)	42.2 (4.2)	264.7 (85.6)	74.3 (16.5)
**Race**										
Han Chinese	1783 (90.0)	776 (28.7)	16.2 (50.3)	115 (67.9)	17.9 (71)	78.5 (22.3)	6.4 (1.7)	42.4 (4.3)	289.4 (90.6)	75.6 (16.2)
Ethnic minorities	148 (7.5)	68 (25.5)	35.5 (114.9)	136.3 (70.3)	27.2 (89.6)	83.5 (36.8)	6.4 (1.9)	39.9 (4.5)	303 (96)	73.4 (18.2)
Missing	50 (2.5)	27 (35.8)	50.8 (143.6)	129.2 (113.3)	44.3 (135.5)	80.2 (25.9)	6.8 (1.4)	41.5 (3.8)	277.4 (80.8)	74.8 (17.5)
**Education**										
No formal education	1200 (60.6)	610 (33.4)	19.8 (64.3)	107.9 (66.6)	23.7 (97.3)	75.5 (21.6)	6.6 (1.9)	41.6 (4.1)	275.6 (92.8)	73.4 (16.6)
Formal education	764 (38.6)	250 (24.7)	17.3 (59.4)	123.9 (70.5)	15.6 (50.7)	81.6 (25.4)	6.3 (1.7)	42.5 (4.6)	301.4 (88.3)	76.9 (16.2)
Missing	17 (0.9)	11 (60.4)	53 (83.7)	123.2 (60.3)	59.5 (87.4)	87.5 (13)	7.3 (1.2)	39.2 (6.2)	290.2 (57.1)	70.5 (11.9)
**Household income (RMB)**										
Tertile 1 (<6,000)	629 (31.8)	233 (25.8)	19 (69.8)	110.5 (68.2)	20.8 (105.6)	76.1 (19.6)	6.5 (1.6)	41.8 (4.1)	270.8 (88.1)	77.9 (15.3)
Tertile 2 (6,000-19,000)	644 (32.5)	278 (29.9)	17.5 (52.4)	120.7 (63.7)	17.9 (56.9)	77.3 (20.6)	6.5 (1.9)	42 (4.3)	286.2 (88.5)	75.8 (16.3)
Tertile 3 (20,000-more than 100,000)	564 (28.5)	283 (28.1)	18.2 (65.7)	121.6 (77.6)	17.2 (53.6)	83.4 (29.7)	6.3 (1.7)	42.7 (4.6)	310.9 (89.7)	73.3 (17.1)
Missing	144 (7.3)	77 (36.5)	22.6 (37.5)	103.4 (49.2)	29.5 (69.1)	79.1 (24.7)	6.5 (1.8)	41.3 (4.5)	302.6 (104.5)	72 (16.7)
**Marital Status**										
Married	772 (39.0)	228 (24.9)	18.6 (66.3)	122.3 (70)	17.7 (54.1)	80.1 (25.3)	6.4 (1.7)	42.6 (4.3)	293.5 (89.4)	77.4 (16)
Separated	40 (2.0)	18 (41.2)	12.4 (40)	124.7 (90)	7.9 (20.1)	83 (19.7)	6.8 (2.1)	38.7 (4.3)	280.8 (82.1)	71.1 (15.3)
Divorced	5 (0.3)	1 (25.3)	12.8 (20.8)	131.7 (110.4)	17.7 (31.7)	70.1 (21.1)	6.5 (1.6)	39.7 (3.8)	271.9 (68.9)	90.7 (17.3)
Widowed	1096 (55.3)	594 (34.1)	18.7 (54.6)	105.3 (63.9)	23 (107.9)	76.2 (21.4)	6.5 (1.8)	41.5 (4.4)	281.5 (90.3)	71.5 (16.6)

Never married	19 (1.0)	6 (38.5)	14.2 (36.3)	115.7 (58.2)	9 (26.8)	83.8 (24.6)	6.5 (1.1)	41.5 (4.9)	321 (143.9)	79.5 (20.1)
Missing	49 (2.5)	24 (34.2)	23.8 (47)	115.4 (70.2)	33.9 (94.7)	77 (23.3)	6.3 (1.8)	41.4 (4)	336.5 (112)	72.1 (16)
**Health condition**										
Very good	102 (5.1)	40 (17.8)	14.8 (25.2)	119.6 (59)	15.5 (34)	78.2 (16.1)	6.1 (1.6)	42.5 (4.1)	327.5 (85.8)	79 (13)
Good	744 (37.6)	282 (22.9)	14.9 (52.9)	120.7 (72.6)	15.6 (89)	78.7 (23.9)	6.6 (1.8)	42.3 (4.6)	289.7 (90.2)	76.9 (16.1)
Fair	750 (37.9)	352 (32.3)	23.9 (73.8)	115.2 (67.4)	23.4 (61.5)	79.4 (25.7)	6.4 (1.7)	42.1 (4.2)	283.1 (85.8)	74.5 (16.9)
Bad	199 (10.0)	103 (43.3)	17.1 (68.7)	108.2 (70.5)	20.5 (59.9)	80.7 (23.4)	6.3 (1.8)	41.8 (4.1)	293.5 (107.4)	71 (17)
Very Bad	12 (0.6)	4 (70.3)	10.3 (20.6)	107.7 (44.2)	14.7 (32.3)	83.8 (16.6)	6.3 (1.5)	42 (5.3)	267.5 (29.5)	62.2 (13.1)
Missing	174 (8.8)	90 (30.7)	22.3 (60.2)	110.1 (57.2)	29.3 (88.5)	73.5 (22.8)	6.7 (1.5)	39.8 (4.7)	305.4 (110.5)	73.8 (16.7)
**Smoking status**										
Never smoker	1432 (72.3)	678 (30.7)	19.4 (65.4)	113.3 (69.2)	19.7 (79.8)	77.5 (24.6)	6.5 (1.7)	42.3 (4.3)	282.5 (88.2)	74.7 (16.6)
Former smoker	161 (8.1)	57 (26.1)	18.9 (44.1)	121.5 (71.9)	24.4 (69.8)	86.1 (23.3)	6.6 (1.9)	41 (4.4)	320.6 (97)	74.4 (16.3)
Current smoker	333 (16.8)	108 (21)	14.5 (53.3)	131.4 (68.1)	14 (49.5)	82.7 (21.5)	6.3 (1.8)	42 (4.6)	306.1 (91.8)	78.6 (15.7)
Missing	55 (2.8)	28 (31.7)	21.1 (44.1)	94.7 (53.9)	32.3 (87)	74.4 (21.4)	6.4 (1.6)	42.5 (4.3)	317.4 (116.6)	74.4 (15.9)
**Drinking status**										
Never drinker	1497 (75.6)	686 (29.7)	17.3 (56.7)	114.8 (69.7)	18.8 (76.5)	77.7 (23.1)	6.5 (1.7)	42.1 (4.3)	281 (88)	75 (16.4)
Former drinker	118 (6.0)	53 (35.5)	41 (130.4)	125.4 (70)	27 (88.1)	89.2 (37.8)	6.6 (1.6)	41.6 (4.6)	326.9 (97.9)	71.8 (19.4)
Current drinker	311 (15.7)	103 (19.1)	14.9 (38.4)	123.8 (65.7)	16.8 (52.9)	81.5 (20.3)	6.1 (1.8)	42.6 (4.4)	315.6 (91.7)	79.2 (14.9)
Missing	55 (2.8)	29 (34.9)	19.1 (42.6)	127.2 (73.7)	26.8 (84.8)	77 (22.8)	6.5 (1.7)	40.2 (5.5)	336.1 (100.6)	74.4 (17.1)
**Physical activity**										
Yes	310 (15.6)	144 (31.5)	14.9 (39.1)	134.9 (90.4)	18.1 (115.5)	80.2 (21.2)	6.3 (1.8)	42 (4.3)	308 (93)	73.8 (15.9)
No	1563 (78.9)	678 (27.9)	19.4 (68.3)	112.1 (62.7)	19.3 (61.5)	78.9 (24.3)	6.5 (1.8)	42.2 (4.5)	285.3 (88.3)	75.7 (16.5)
Missing	108 (5.5)	49 (26.1)	17.5 (30.2)	120.1 (59.7)	20.2 (48.5)	76.9 (28.3)	6.5 (1.5)	41.8 (3.9)	296.8 (107)	77.3 (16.9)
**Body mass index (kg/m^2)**										
Underweight (<18.5)	466 (23.5)	264 (38.2)	16.6 (39.9)	106.1 (57.8)	23.2 (143.8)	80.8 (24.9)	6.2 (1.9)	41 (4.5)	274.7 (85.4)	72.2 (18.2)
Normal (18.5-24.9)	1135 (57.3)	456 (27.8)	17.5 (64.2)	118.5 (72.6)	16.9 (52.3)	78.9 (23.7)	6.5 (1.7)	42 (4.2)	284.4 (93.3)	75.6 (16.1)
Overweight (25.0-29.9)	228 (11.5)	73 (25.2)	21.9 (63.5)	121.5 (67.2)	22.3 (65.9)	79.5 (25.5)	6.5 (1.8)	43.5 (4.8)	309.9 (84.4)	76.3 (16.3)
Obese (>=30)	56 (2.8)	22 (20.2)	20.9 (62)	104.2 (55.5)	26.8 (100)	74.2 (19.2)	6.2 (1.5)	42.2 (4.1)	327.4 (81.6)	78.1 (14.3)
Missing	96 (4.8)	56 (41)	20.5 (70.1)	123.9 (60.6)	21.5 (59.3)	76.9 (23.2)	6.7 (1.6)	40.6 (4.7)	319.1 (80.9)	77.5 (18.3)
**Hypertension**										

Yes	1122 (56.6)	544 (33.4)	24.3 (75.2)	113.2 (65.5)	25.4 (96.1)	80.6 (25.4)	6.4 (1.8)	42.4 (4.4)	296.7 (88.5)	73.9 (16.3)
No	839 (42.4)	315 (23.2)	12.1 (40.8)	120.7 (72.7)	12.6 (39.2)	77.3 (22.4)	6.5 (1.7)	41.9 (4.4)	283.4 (92.8)	77.1 (16.3)
Missing	20 (1.0)	12 (33.6)	28 (110.9)	158 (83.4)	15.6 (60)	87.2 (25.8)	7.1 (1.5)	37.1 (3)	338.1 (115.2)	70.7 (19.4)
**Diabetes**										
Yes	48 (2.4)	22 (28.8)	61.3 (161.6)	128.1 (67.8)	43 (103)	85.4 (41.9)	6.4 (1.4)	41.8 (4)	303.6 (82.1)	73.4 (18.9)
No	1902 (96.0)	831 (28.3)	15.9 (50.1)	116.3 (69.2)	17.7 (72.2)	78.6 (22.7)	6.4 (1.8)	42.2 (4.4)	289.7 (91.7)	75.6 (16.3)
Missing	31 (1.6)	18 (50.3)	64.5 (108.5)	153.8 (76.2)	55.4 (82.8)	88.7 (22.6)	7.1 (2.6)	38.5 (5)	299.8 (54.1)	61.4 (17.5)
