# Supplementary material for: Chronic kidney disease biomarkers and mortality among older adults: A comparison study of survey samples in China and the United States
Source: PLoS One. 2022 Jan 12;17(1):e0260074. doi: 10.1371/journal.pone.0260074 (PMC8754291; doi:10.1371/journal.pone.0260074)
Supplement: S7 Table — 1. Demographic characteristics and weighted mean (SD) of biomarkers (Chinese participants: CLHLS 2012). 2. Demographic characteristics and weighted mean (SD) of biomarkers (US participants: NHANES 2011–2014). (ZIP) [file pone.0260074.s007.zip › S7-2 Table.pdf]

S7-2 Table. Demographic characteristics and weighted mean (SD) of biomarkers (US participants: NHANES 2011-2014)

Characteristics	n (%)	CKD n (%)	Urine microalbumin (mg/L)	Urinary creatinine (mg/dL)	Albumin creatinine ratio (mg/g)	Serum creatinine (μmol/L)	Blood urea nitrogen (mmol/L)	Plasma albumin (g/L)	Uric acid (μmol/L)	eGFR (mL/min per 1.73 m^2)
**Total**	2177 (100)	921 (38.9)	49.8 (246.8)	103.9 (65.0)	57.9 (328.4)	88.4 (34.9)	6.1 (2.6)	41.9 (2.9)	335.4 (87.2)	69.9 (18.1)
**Age (mean ± SD)**	72.9±0.2	75.0±0.2								
**Age group**										
65-69	682 (33.4)	187 (22.6)	34.0 (189.4)	111.3 (72.5)	36.8 (306.2)	83.4 (32.0)	5.6 (2.2)	42.2 (2.8)	331.2 (87.9)	77.3 (16.1)
70-74	567 (26.6)	202 (34.9)	32.3 (143.2)	100.4 (60.3)	30.4 (118.1)	84.9 (28.6)	5.7 (2.0)	42.3 (2.9)	333.5 (80.6)	72.0 (16.2)
75-79	361 (16.4)	179 (46.0)	64.0 (298.7)	103.6 (63.1)	74.4 (351.5)	92.4 (38.3)	6.3 (2.6)	41.9 (2.8)	339.8 (91.4)	65.8 (18.0)
80+	567 (23.6)	353 (61.8)	82.4 (347.2)	97.5 (59.1)	107.7 (467.8)	96.9 (40.7)	7.1 (3.1)	41.3 (3.1)	340.6 (89.7)	60.0 (17.5)
**Gender**										
Male	1072 (45.6)	457 (37.4)	63.5 (293.1)	125.0 (67.4)	71.2 (422.4)	100.2 (39.4)	6.3 (2.6)	42.2 (3.0)	357.6 (80.9)	69.6 (17.4)
Female	1105 (54.4)	464 (40.2)	38.4 (199.2)	86.2 (57.3)	46.8 (220.0)	78.6 (27.0)	5.9 (2.5)	41.7 (2.9)	316.9 (87.9)	70.1 (18.6)
**Race/Ethnicity**										
Mexican American	169 (3.3)	58 (34.4)	120.1 (438.8)	109.1 (68.8)	154.7 (577.6)	84.6 (45.5)	5.9 (2.8)	41.8 (3.3)	318.7 (82.5)	75.6 (19.6)
Other Hispanics	188 (3.6)	69 (37.5)	72.5 (214.7)	106.6 (58.3)	58.9 (153.1)	83.5 (31.3)	5.9 (2.5)	41.6 (3.1)	324.7 (80.6)	72.3 (18.4)
Non-Hispanic White	1151 (79.6)	517 (38.5)	37.8 (181.3)	101.8 (62.9)	46.1 (269.7)	87.6 (31.6)	6.2 (2.5)	42.0 (2.9)	333.5 (86.4)	69.3 (17.4)
Non-Hispanic Black	439 (7.7)	195 (45.2)	121.2 (516.7)	134.7 (84.4)	114.9 (476.4)	103.2 (54.5)	5.8 (3.0)	41.2 (3.2)	361.3 (91.4)	70.7 (22.4)
Non-Hispanic Asian	196 (4.0)	64 (32.6)	80.1 (358.6)	84.1 (51.6)	113.3 (701.5)	80.8 (38.0)	5.7 (2.1)	42.6 (2.9)	340.0 (82.0)	75.0 (16.7)
Other races	34 (1.8)	18 (56.3)	34.0 (110.6)	90.5 (52.4)	33.0 (97.4)	94.4 (27.9)	5.6 (2.5)	42.0 (3.2)	354.0 (108.9)	67.6 (20.4)
**Education**										
Below high school	649 (20.5)	318 (50.5)	78.6 (342.2)	108.2 (70.2)	89.8 (365.5)	94.1 (42.7)	6.4 (3.3)	41.6 (3.2)	348.8 (90.9)	67.1 (20.8)
High school	504 (22.5)	213 (38.7)	46.4 (194.0)	107.5 (67.2)	51.4 (233.9)	87.9 (33.7)	5.9 (2.3)	41.9 (3.0)	336.1 (85.1)	69.2 (17.4)
College or above	1019 (56.9)	386 (34.7)	40.8 (222.7)	100.8 (62.0)	49.0 (345.3)	86.6 (32.0)	6.1 (2.3)	42.1 (2.8)	330.3 (86.1)	71.2 (17.1)
Missing	5 (0.1)	4 (83.4)	54.8 (44.8)	158.6 (59.9)	49.2 (61.4)	93.8 (21.0)	4.1 (1.7)	41.2 (1.3)	354.6 (72.4)	61.0 (17.6)
**Income (PIR)**										
Tertile 1 (0-1.87)	928 (30.7)	416 (44.8)	66.3 (315.1)	100.3 (67.4)	74.8 (353.6)	89.7 (37.8)	6.1 (2.9)	41.6 (3.0)	339.6 (89.8)	68.6 (19.8)
Tertile 2 (1.88-3.86)	582 (30.5)	251 (39.0)	45.8 (204.4)	107.5 (66.6)	56.5 (302.9)	88.9 (36.1)	6.1 (2.3)	41.8 (3.0)	335.4 (84.8)	69.0 (17.6)
Tertile 3 (>=3.87)	474 (31.5)	176 (33.8)	33.6 (157.9)	104.3 (62.3)	35.6 (262.9)	87.5 (29.0)	6.0 (2.3)	42.2 (2.8)	334.7 (87.0)	71.4 (16.7)
Missing	193 (7.3)	78 (35.7)	68.0 (364.1)	101.9 (58.5)	89.2 (515.7)	85.3 (40.2)	6.5 (2.9)	42.5 (3.1)	321.2 (84.5)	72.4 (17.5)

**Marital Status**										
Married	1173 (59.6)	450 (35.0)	49.5 (252.9)	106.9 (65.4)	59.2 (371.7)	88.9 (35.2)	6.0 (2.5)	42.2 (2.7)	336.8 (85.2)	70.9 (17.1)
Separated	44 (0.9)	15 (37.5)	164.1 (376.9)	103.3 (65.1)	217.2 (594.4)	88.6 (34.3)	6.3 (3.2)	41.7 (3.2)	335.7 (93.3)	0.3 (19.5)
Divorced	258 (10.9)	112 (39.6)	34.4 (130.1)	107.2 (69.2)	35.7 (146.9)	86.0 (31.6)	5.8 (2.4)	41.9 (2.8)	334.7 (95.5)	70.6 (19.1)
Widowed	562 (22.8)	292 (50.2)	53.4 (256.3)	92.4 (61.0)	61.4 (255.7)	87.4 (35.0)	6.4 (2.8)	41.3 (3.4)	332.2 (87.2)	66.3 (19.3)
Never married	100 (3.5)	40 (36.9)	74.7 (359.7)	102.5 (63.5)	73.1 (369.0)	94.9 (44.1)	6.2 (2.5)	41.9 (2.9)	328.0 (86.9)	69.4 (20.0)
Living with partner	39 (2.3)	11 (28.3)	14.2 (15.5)	125.3 (59.6)	11.5 (14.2)	86.9 (22.6)	5.9 (1.4)	43.1 (2.8)	346.9 (90.9)	76.0 (16.2)
Missing	1 (0.0)	1 (100.0)	14.6 (0)	154 (0)	9.5 (0)	80.4 (0)	6.8 (0)	32.0 (0)	410.4 (0)	59.8 (0)
**Health condition**										
Excellent	158 (9.5)	59 (36.1)	20.0 (59.3)	102.1 (61.7)	25.6 (94.8)	87.4 (27.6)	6.1 (2.1)	42.0 (2.9)	335.5 (80.0)	69.8 (15.1)
Very good	515 (28.6)	172 (30.1)	25.0 (119.7)	101.6 (61.1)	24.7 (91.9)	81.5 (21.7)	5.9 (2.0)	42.3 (2.9)	320.4 (84.8)	73.0 (16.0)
Good	811 (37.7)	336 (38.1)	53.1 (262.0)	103.1 (64.6)	59.8 (324.7)	88.7 (31.2)	6.0 (2.4)	42.0 (2.8)	340.4 (82.4)	69.8 (17.9)
Fair	509 (17.5)	264 (53.9)	89.0 (366.7)	108.3 (72.6)	108.5 (483.8)	97.1 (49.0)	6.5 (3.2)	41.4 (3.2)	348.6 (98.3)	66.3 (20.7)
Poor	95 (3.4)	49 (50.8)	115.7 (422.4)	107.5 (69.4)	156.8 (801.2)	98.2 (65.4)	6.1 (3.2)	40.8 (3.7)	338.0 (95.2)	66.9 (22.9)
Missing	89 (3.3)	41 (40.9)	38.0 (103.3)	109.9 (63.3)	47.8 (170.3)	92.4 (39.5)	6.6 (4.2)	41.8 (2.6)	335.9 (91.1)	67.0 (20.0)
**Smoking status**										
Never smoker	1096 (49.4)	445 (37.1)	46.8 (261.3)	99.6 (62.3)	55.2 (344.9)	86.6 (36.2)	6.1 (2.5)	41.9 (2.9)	327.5 (84.6)	69.5 (17.9)
Former smoker	857 (41.6)	379 (41.2)	46.9 (205.3)	108.3 (66.3)	52.5 (263.5)	89.9 (30.0)	6.1 (2.4)	42.0 (2.9)	343.2 (90.2)	69.7 (17.7)
Current smoker	222 (8.9)	96 (38.4)	80.0 (326.6)	106.4 (71.8)	98.3 (472.8)	92.0 (46.7)	5.6 (3.4)	41.7 (3.1)	343.4 (82.9)	72.8 (20.6)
Missing	2 (0.1)	1 (76.7)	92.6 (44.2)	225.7 (31.3)	38.6 (17.3)	83.6 (0.7)	6.7 (0.8)	42.1 (1.7)	331.8 (30.2)	65.4 (15.7)
**Drinking status**										
Never drinker	392 (14.7)	177 (48.1)	71.8 (375.9)	97.5 (66.6)	93.5 (460.0)	90.2 (49.8)	6.3 (3.0)	41.6 (2.9)	333.1 (91.4)	66.8 (20.2)
Former drinker	318 (14.3)	153 (46.2)	72.7 (266.5)	99.1 (60.4)	83.8 (319.0)	87.1 (31.7)	6.1 (2.8)	41.5 (2.9)	330.6 (87.4)	68.1 (19.4)
Current drinker	1356 (67.0)	541 (35.2)	41.0 (209.7)	106.0 (65.7)	45.5 (301.4)	88.1 (31.3)	6.0 (2.2)	42.1 (2.9)	337.1 (86.0)	71.1 (17.0)
Missing	111 (4.0)	50 (41.3)	35.7 (95.1)	109.3 (60.9)	43.4 (155.5)	91.8 (37.3)	6.6 (4.0)	41.7 (2.9)	334.0 (88.8)	67.0 (19.7)
**Physical activity**										
Yes	868 (41.2)	324 (33.2)	43.2 (225.8)	103.5 (65.9)	52.2 (369.8)	85.8 (35.1)	5.9 (2.3)	42.3 (2.7)	335.0 (86.6)	72.8 (17.2)
No	1306 (58.6)	596 (43.0)	54.4 (260.6)	104.2 (64.5)	62.0 (296.1)	90.3 (34.7)	6.2 (2.7)	41.7 (3.0)	335.8 (87.7)	67.9 (18.4)
Missing	3 (0.2)	1 (14.9)	90.4 (209.0)	92.3 (32.5)	55.8 (124.4)	85.7 (7.2)	5.2 (0.8)	39.1 (2.5)	329.1 (39.1)	76.9 (6.1)
**Body mass index (kg/m^2)**										

Underweight (<18.5)	36 (1.8)	16 (40.1)	75.1 (253.2)	93.6 (60.8)	82.0 (230.9)	74.6 (19.8)	5.5 (2.2)	43.0 (4.2)	261.3 (76.9)	77.1 (15.9)
Normal (18.5-24.9)	579 (26.4)	228 (35.1)	50.7 (266.3)	92.2 (61.6)	68.3 (428.7)	85.4 (38.1)	6.0 (2.5)	42.3 (2.8)	309.9 (81.2)	71.5 (18.1)
Overweight (25.0-29.9)	776 (36.1)	316 (36.7)	29.8 (114.3)	102.9 (62.3)	33.9 (143.5)	89.0 (28.9)	6.0 (2.3)	42.2 (2.7)	333.6 (84.4)	69.6 (17.3)
Obese (>=30)	746 (33.9)	335 (42.9)	67.5 (323.8)	113.7 (67.9)	71.6 (381.2)	90.2 (37.2)	6.1 (2.7)	41.3 (3.0)	359.4 (87.3)	68.8 (18.4)
Missing	40 (1.8)	26 (61.2)	82.0 (168.0)	119.3 (82.9)	105.2 (270.5)	102.4 (51.0)	7.4 (3.8)	40.5 (3.2)	366.6 (79.3)	64.5 (24.7)
**Hypertension**										
Yes	746 (30.8)	371 (47.0)	88.3 (385.9)	95.5 (62.3)	113.5 (537.1)	90.3 (41.8)	6.2 (2.8)	42.0 (3.1)	333.7 (87.1)	68.0 (19.2)
No	1431 (69.2)	550 (35.3)	32.7 (144.2)	107.6 (65.9)	33.2 (159.3)	87.6 (31.3)	6.0 (2.4)	41.9 (2.8)	336.2 (87.2)	70.7 (17.5)
**Diabetes**										
Yes	526 (20.0)	302 (55.7)	129.2 (471.1)	110.7 (67.0)	161.9 (657.0)	100.3 (51.7)	6.8 (3.2)	41.3 (2.9)	352.2 (93.9)	65.3 (21.0)
No	1650 (80.0)	618 (34.7)	30.0 (136.7)	102.2 (64.4)	31.9 (153.1)	85.5 (28.5)	5.9 (2.3)	42.1 (2.9)	331.2 (84.9)	71.1 (17.0)
Missing	1 (0.0)	1 (100.0)	3.2 (0)	74.0 (0)	43 (0)	99.9 (0)	7.1 (0)	37.0 (0)	404.5 (0)	47.0 (0)
