# Supplementary material for: Chronic kidney disease biomarkers and mortality among older adults: A comparison study of survey samples in China and the United States
Source: PLoS One. 2022 Jan 12;17(1):e0260074. doi: 10.1371/journal.pone.0260074 (PMC8754291; doi:10.1371/journal.pone.0260074)
Supplement: S8 Table — (PDF) [file pone.0260074.s008.pdf]

S8 Table. Odds ratio (95% CI) of factors associated with CKD in Chinese and US population (weighted).

CLHLS				NHANES			
**Characteristics**	**n (%)**	**OR (95% CI) ***	**P value**	**Characteristics**	**n (%)**	**OR (95% CI) ***	**P value**
Total	1981 (100)			Total	2177 (100)		
Age (mean ± SD)	85.3±12.0	\	\	Age (mean ± SD)	72.9±0.2	\	\
**Age group**				**Age group**			
65-69	240 (12.1)	Ref	\	65-69	682 (33.4)	Ref	\
70-74	240 (12.1)	0.95 (0.53, 1.68)	0.854	70-74	567 (26.6)	**1.91 (1.36, 2.69)**	**<0.001**
75-79	217 (11.0)	**2.23 (1.26, 3.99)**	**0.006**	75-79	361 (16.4)	**2.80 (1.93, 4.08)**	**<0.001**
80+	1284 (64.8)	**3.81 (2.11, 6.97)**	**<0.001**	80+	567 (23.6)	**5.99 (4.22, 8.50)**	**<0.001**
**Gender**				**Gender**			
Male	928 (46.8)	Ref	\	Male	1072 (45.6)	Ref	\
Female	1053 (53.2)	1.25 (0.76, 2.05)	0.379	Female	1105 (54.4)	1.06 (0.82, 1.38)	0.668
**Race**				**Race/Ethnicity**			
Han Chinese	1783 (90.0)	Ref	\	Mexican American	169 (3.3)	Ref	\
Ethnic minorities	148 (7.5)	0.83 (0.39, 1.67)	0.611	Other Hispanics	188 (3.6)	1.11 (0.66, 1.84)	0.701
Missing	50 (2.5)	1.10 (0.20, 4.96)	0.901	Non-Hispanic White	1151 (79.6)	1.51 (0.98, 2.34)	0.063
				Non-Hispanic Black	439 (7.7)	**1.59 (1.02, 2.50)**	**0.043**
				Non-Hispanic Asian	196 (4.0)	1.18 (0.67, 2.07)	0.569
				Other races	34 (1.8)	**3.11 (1.10, 8.79)**	**0.033**
**Education**				**Education**			
No formal education	1200 (60.6)	Ref	\	Below high school	649 (20.5)	Ref	\
Formal education	764 (38.6)	1.21 (0.76, 1.94)	0.437	High school	504 (22.5)	**0.69 (0.49, 0.96)**	**0.027**
Missing	17 (0.9)	3.51 (0.08, 299.88)	0.476	College or above	1019 (56.9)	0.73 (0.53, 1.01)	0.055
				Missing	5 (0.1)	3.05 (0.24, 39.27)	0.392
**Household income (RMB)**				**Income (PIR)**			
Tertile 1 (<6,000)	629 (31.8)	Ref	\	Tertile 1 (0-1.87)	928 (30.7)	Ref	\
Tertile 2 (6,000-19,000)	644 (32.5)	1.26 (0.76, 2.10)	0.374	Tertile 2 (1.88-3.86)	582 (30.5)	1.06 (0.79, 1.42)	0.695

Tertile 3 (20,000-over 100,000)	564 (28.5)	1.29 (0.76, 2.21)	0.345
Missing	144 (7.3)	1.32 (0.47, 3.57)	0.584
**Marital Status**			
Married	772 (39.0)	Ref	\
Not married	1160 (58.5)	0.99 (0.63, 1.56)	0.978
Missing	49 (2.5)	1.73 (0.10, 18.75)	0.670
**Health condition**			
Very good	102 (5.1)	Ref	\
Good	744 (37.6)	1.09 (0.45, 2.96)	0.861
Fair	750 (37.9)	1.65 (0.67, 4.54)	0.302
Bad/Very bad	211 (10.6)	2.76 (1.01, 8.31)	0.057
Missing	174 (8.8)	0.50 (0.05, 3.68)	0.516
**Smoking status**			
Never smoker	1432 (72.3)	Ref	\
Former smoker	161 (8.1)	0.72 (0.29, 1.71)	0.470
Current smoker	333 (16.8)	0.85 (0.45, 1.57)	0.610
Missing	55 (2.8)	0.71 (0.06, 4.93)	0.741
**Drinking status**			
Never drinker	1497 (75.6)	Ref	\
Former drinker	118 (6.0)	1.44 (0.61, 3.29)	0.391
Current drinker	311 (15.7)	0.70 (0.35, 1.36)	0.300
Missing	55 (2.8)	1.45 (0.15, 10.27)	0.720
**Physical activity**			
Yes	310 (15.6)	Ref	\
No	1563 (78.9)	0.71 (0.43, 1.20)	0.199
Missing	108 (5.5)	0.74 (0.27, 1.89)	0.540
**Body mass index (kg/m^2)**			
Underweight (<18.5)	466 (23.5)	Ref	\

Tertile (>=3.87)	474 (31.5)	1.26 (0.90, 1.77)	0.182
Missing	193 (7.3)	0.83 (0.55, 1.25)	0.368
**Marital Status**			
Married	1173 (59.6)	Ref	\
Not married	1003 (40.4)	1.20 (0.93, 1.55)	0.172
Missing	1 (0.0)	NA	<0.001
**Health condition**			
Excellent	158 (9.5)	Ref	\
Very good	515 (28.6)	0.63 (0.38, 1.05)	0.077
Good	811 (37.7)	0.80 (0.49, 1.32)	0.383
Fair/Poor	604 (20.9)	1.32 (0.78, 2.23)	0.304
Missing	89 (3.3)	1.30 (0.36, 4.61)	0.689
**Smoking status**			
Never smoker	1096 (49.4)	Ref	\
Former smoker	857 (41.6)	1.29 (0.98, 1.69)	0.071
Current smoker	222 (8.9)	**1.61 (1.03, 2.52)**	**0.037**
Missing	2 (0.1)	1.79 (0.20, 15.69)	0.600
**Drinking status**			
Never drinker	392 (14.7)	Ref	\
Former drinker	318 (14.3)	0.99 (0.66, 1.49)	0.951
Current drinker	1356 (67.0)	0.76 (0.53, 1.08)	0.122
Missing	111 (4.0)	0.48 (0.16, 1.43)	0.189
**Physical activity**			
Yes	868 (41.2)	Ref	\
No	1306 (58.6)	1.05 (0.82, 1.35)	0.710
Missing	3 (0.2)	0.39 (0.04, 4.19)	0.434
**Body mass index (kg/m^2)**			
Underweight (<18.5)	36 (1.8)	Ref	\

Normal (18.5-24.9)	1135 (57.3)	0.79 (0.43, 1.45)	0.435	Normal (18.5-24.9)	579 (26.4)	1.02 (0.43, 2.44)	0.958
Overweight (25.0-29.9)	228 (11.5)	0.75 (0.36, 1.58)	0.446	Overweight (25.0-29.9)	776 (36.1)	1.18 (0.49, 2.80)	0.715
Obese (>=30)	56 (2.8)	0.58 (0.16, 1.85)	0.380	Obese (>=30)	746 (33.9)	1.50 (0.62, 3.62)	0.364
Missing	96 (4.8)	1.55 (0.29, 7.61)	0.596	Missing	40 (1.8)	2.15 (0.67, 6.84)	0.197
**Hypertension**				**Hypertension**			
Yes	1122 (56.6)	Ref	\	Yes	746 (30.8)	Ref	\
No	839 (42.4)	**0.64 (0.43, 0.97)**	**0.034**	No	1431 (69.2)	**0.74 (0.57, 0.94)**	**0.015**
Missing	20 (1.0)	0.56 (0.02, 7.19)	0.666				
**Diabetes**				**Diabetes**			
Yes	48 (2.4)	Ref	\	Yes	526 (20.0)	Ref	\
No	1902 (96.0)	1.04 (0.42, 2.83)	0.933	No	1650 (80.0)	**0.45 (0.34, 0.61)**	**<0.001**
Missing	31 (1.6)	1.70 (0.11, 26.74)	0.687	Missing	1 (0.0)	**NA**	**<0.001**

Abbreviations: OR = odds ratio, CI = confidence interval, CKD = chronic kidney diseases, eGFR = estimated glomerular filtration rate, PIR = ratio of family income to poverty.
* The multi-variate analysis contained all the variables listed above in the logistic regression models.
