# Supplementary material for: Chronic kidney disease biomarkers and mortality among older adults: A comparison study of survey samples in China and the United States
Source: PLoS One. 2022 Jan 12;17(1):e0260074. doi: 10.1371/journal.pone.0260074 (PMC8754291; doi:10.1371/journal.pone.0260074)
Supplement: S9 Table — (PDF) [file pone.0260074.s009.pdf]

S9 Table. Hazard ratio (95% CI) of CKD biomarkers on mortality in Chinese and US population (weighted).

Model	Factor	CLHLS			NHANES		
		Crude	Age-sex adjusted	All covariates adjusted †	Crude	Age-sex adjusted	All covariates adjusted †
Model A	Urine microalbumin (mg/L)	**1.003 (1.002, 1.004)***	1.002 (1.000, 1.003)	1.001 (1.00, 1.002)	**1.001 (1.001, 1.001)***	**1.00 (1.00, 1.00)***	**1.00 (1.00, 1.00)***
Model B	Urinary creatinine (mg/dL)	0.998 (0.996, 1.001)	0.999 (0.997, 1.001)	0.999 (0.996, 1.001)	**0.999 (0.999, 0.999)***	**1.00 (1.00, 1.00)***	**0.999 (0.999, 0.999)***
Model C	Blood urea nitrogen (mmol/L)	**1.13 (1.04, 1.23)**	1.05 (0.97, 1.13)	1.06 (0.99, 1.14)	**1.17 (1.17, 1.17)***	**1.12 (1.12, 1.13)***	**1.11 (1.10, 1.11)***
Model D	Plasma albumin (g/L)	**0.92 (0.89, 0.95)***	**0.95 (0.92, 0.98)**	**0.96 (0.93, 0.99)**	**0.85 (0.85, 0.85)***	**0.87 (0.87, 0.87)***	**0.89 (0.89, 0.89)***
Model E	Uric acid (umol/L)	1.002 (1.000, 1.003)	1.00 (0.998, 1.002)	1.001 (0.999, 1.002)	**1.003 (1.003, 1.003)***	**1.003 (1.003, 1.003)***	**1.002 (1.002, 1.003)***
Model F	**Albumin creatinine ratio (mg/g)**	**1.001 (1.001, 1.001)***	**1.00 (1.00, 1.001)**	**1.00 (1.00, 1.001)***	**1.000 (1.000, 1.000)***	**1.00 (1.00, 1.00)***	**1.00 (1.00, 1.00)***
Model G	**Serum creatinine (μ mol/L)**	**1.01 (1.006, 1.014)***	1.005 (0.999, 1.01)	1.004 (1.00, 1.008)	**1.006 (1.006, 1.006)***	**1.006 (1.006, 1.006)***	**1.005 (1.005, 1.005)***
Model H	**eGFR**	**0.98 (0.97, 0.98)***	0.995 (0.98, 1.006)	0.997 (0.988, 1.006)	**0.97 (0.97, 097)***	**0.98 (0.98, 0.98)***	**0.99 (0.99, 0.99)***
Model I	**CKD**						
	No	Ref	Ref	Ref	Ref	Ref	Ref
	Yes	**1.96 (1.42, 2.71)***	1.20 (0.87, 1.66)	1.09 (0.81, 1.46)	**4.06 (4.05, 4.07)***	**2.60 (2.59, 2.60)***	**2.17 (2.17, 2.18)***
Model J	**Categorical eGFR**						
	<30	**8.01 (3.17, 20.26)***	2.23 (0.85, 5.88)	**2.24 (1.07, 4.69)***	**9.78 (9.72, 9.83)***	**3.52 (3.50, 3.54)***	2.27 (0.92, 5.62)
	30~	**5.08 (2.43, 10.60)***	1.17 (0.57, 2.39)	1.29 (0.60, 2.76)	**6.64 (6.61, 6.67)***	**2.14 (2.13, 2.15)***	1.88 (0.86, 4.11)
	45~	**2.99 (1.62, 5.51)***	0.97 (0.52, 1.81)	0.92 (0.48, 1.77)	**2.51 (2.49, 2.52)***	**0.93 (0.93, 0.94)***	0.98 (0.45, 2.12)
	60~	**2.02 (1.17, 3.48)***	0.94 (0.54, 1.64)	1.02 (0.57, 1.83)	**1.48 (1.47, 1.48)***	**0.78 (0.77, 0.78)***	0.86 (0.41, 1.75)

90~	Ref	Ref	Ref	Ref	Ref	Ref

Abbreviations: CI = confidence interval, eGFR = estimated glomerular filtration rate, CKD = chronic kidney diseases.

*** $p<0.001$, **$p<0.01$, *$p<0.05$.

† Adjusted for age, gender, race, educational level, income, marital status, health condition, smoking status, drinking status, physical activity, body mass index, hypertension and diabetes.

References

1. Levey AS, Stevens LA, Schmid CH, Zhang YL, Castro AF, Feldman HI, et al. A new equation to estimate glomerular filtration rate. Annals of internal medicine. 2009;150(9):604-12.
