# Supplementary material for: Chronic kidney disease biomarkers and mortality among older adults: A comparison study of survey samples in China and the United States
Source: PLoS One. 2022 Jan 12;17(1):e0260074. doi: 10.1371/journal.pone.0260074 (PMC8754291; doi:10.1371/journal.pone.0260074)
Supplement: Attachment — Submitted filename: rebuttal letter.docx [file pone.0260074.s011.docx]

1. Regarding the extended tables, please consider to use bold style to underline the statistically significant results (this is just a suggestion, not obligatory requirement).

***Response: We thank the editor for pointing this out. We have bolded all the statistically significant results in all tables, including supplementary tables.***

2. Please use 2 decimal signs in OR, i.e. instead of "1.801 (1.054-3.075)" use "1.80 (1.05-3.07)"; except of the cases where more decimal signs are required to demonstrate the 95%CI close to 1 (like "1.0003-1.18").

***Response: We used two significant digits and revised all the numbers in the tables, including supplementary tables.***

3. Please revise the manuscript for style describing intervals, and change it to more easier to readers (i.e. avoid technical expressions like "eGFR in [30,45) did not increase mortality risk", and use instead "eGFR 30-45 ml/min/1.73m2")

***Response: We thank the editor for this comment. We have revised accordingly in Line 476:***

***“… while eGFR of 30-45 mL/min per 1.73 m^2^ did not increase mortality risk significantly …”***

4. Considering the reviewer's comment "Positive correlations should be assessed for both statistical significance and clinical relevance. The reported OR of 1.000 (1.000, 1.000) in line 351 is apparently statistically significant (with more decimal places?) but is meaningless clinically." and your response "...At a later stage, we will work with the editorial staff to ensure that the reporting guidelines are met....." - Please consider that once the manuscript is accepted for the publication, there will be only minimal grammar/style changes implemented by the Editorial office. Thus, all issues should be resolved during the revision, and this particular reviewer's comment should be addressed properly now, before the "Accepted" decision.

***Response: We thank the editor for the reminding. We revised the phrases as below (Line 456-459):***

***“The association between urinary albumin and mortality was statistically significant but we note it is not clinically meaningful because of low effect estimate values [HR (95%CI): 1.001 (1.000, 1.002) in CLHLS, 1.000 (1.000, 1.000) in NHANES]. ”***

5. The Associate Editor reviewed your manuscript and has indicated "Please assess the reporting of this observational study of clinical data using the STROBE checklist ([http://www.strobe-statement.org)"](https://urldefense.com/v3/__http:/www.strobe-statement.org)*22__;JQ!!OToaGQ!4C0JGqi5E3E-SvIHplUXvIRAV0QoPFcayj4AP56bqDKjHQIwDzRRnAcZwEUo6IGi5torTOY$)

***Response: We appreciate this feedback from the Associate Editor. We have evaluated the manuscript based on the STROBE checklist point by point, and found all requirements were met. We attached a checklist in supplementary files.***

6. Please use in the "Article summary" the phrases that a reader could understand without reading the manuscript. For example, the phrase "CKD is associated with higher mortality in US study participants, perhaps due to comorbid conditions." would be more clear if changed to something like "CKD is associated with higher mortality in US but not in China study participants, perhaps due to comorbid conditions." Instead of general statement "US study participants had higher concentrations of CKD-related biomarker levels compared with Chinese participants" use the complete list of biomarkers.
The item "Strengths and limitations of this study. Our data was derived from large nationally-representative cohorts in both countries. However, there are challenges in age distribution differences, standardization of CKD biomarker measurement techniques, as well as covariates definitions." should be rephrased for clarity and conciseness.

***Response: We appreciate the editor for this helpful comment. We have rephrased as below in Line 58-66:***

- ***Compared to Chinese participants, people in the US study had higher concentrations of CKD-related biomarker, including urinary albumin, urinary creatinine, ACR, serum creatinine, plasma albumin, uric acid and eGFR.***
- ***CKD was associated with higher mortality in US but not in China study participants, perhaps due to unadjusted comorbid conditions.***
- ***Strengths and limitations of this study. Our data was derived from large nationally-representative cohorts in both countries. However, the findings may be limited by the differences of age distribution, biomarker measurement techniques and covariates definitions between the two national datasets.***

7. The description of two groups comparison like "The urinary albumin in China was lower than in the US (mean: 25.0 vs. 76.4, median: 5 vs. 11.2, IQR: 17.8 vs. 11.2 unit: mg/L)" or "people with lower education (27.2 mg/L of “no formal education” vs 103.9 mg/L of “below high school”)" has limited meaning. Please use plain language and refer to the table with numeric results, or indicate widely accepted measures of central tendency and variability in the text. This also concerns the description at lines 313-339.

***Response: We thank the editor for this helpful comment. We have rewritten the three paragraphs as mentioned (Line 332-382):***

***“There was a large difference in the urinary albumin between China and the US. The mean of urinary albumin level in China was 25.0 mg/L (SD: 75.4), much lower than that in the US (76.4 mg/L, SD: 359.6). In China, higher urinary albumin level usually appeared along with factors like age over 80 (29.5 mg/L), female (25.8 mg/L), high household income (29.8 mg/L), widowed (28.6 mg/L), good health condition (25.7 mg/L), never smoker (26.6 mg/L), and former drinker (34.7 mg/L) (Table 1-1). However, in the US, people had higher urinary albumin were more likely to be aged 75-79 (109.3 mg/L), male (98.1 mg/L), with low household income (88.8 mg/L), separated (134.2 mg/L), with poor health condition (171.8 mg/L), current smoker (111.6 mg/L), and never drinker (98.0 mg/L) (Table 1-2). Besides, some characteristics were found related to higher urinary albumin level in both countries, including lower education, obese, insufficient physical activity, hypertension, and diabetes (Table 1-1 & 1-2).***

***In addition to urinary albumin, the mean level of ACR in Chinese participants (41.7 mg/g, SD: 231.5) was also significantly lower than that in the US (85.0 mg/g, SD: 449.3). In both countries, the subgroups with the lowest ACR were those were aged 70-74, with formal education, drinking currently, overweight, or without hypertension. However, we did not find a similar pattern in the two countries in terms of the relationship between ACR and other demographic or lifestyle characteristics.***

***Moreover, Chinese participants (292.9 μmol/L, SD: 90.4) also showed a lower mean level of uric acid than the US (341.3 μmol/L, SD: 88.0). Besides, male presented a higher mean level than female in both Chinese (male: 314.8 μmol/L, female: 274.0 μmol/L) and the US participants (male: 361.1 μmol/L, female: 322.0 μmol/L). Also, people who were younger, never smoker, never drinker, not taking physical activity, underweight, without diabetes tended to have lower uric acid in both CLHLS and NHANES group.”***

8. Some references have no page numbers or eNumber, or ISBN (where appropriate) - like ref 22, 26 and many others. Please check all references and format appropriately.

***Response: We thank the editor for pointing this out. We checked all references and reformatted references no. 3, 4, 10, 12, 17, 22, and 26.***

9. It seems the Table 2 title should be "Odds ratio (95% CI) of factors associated with CKD in CLHLS 2011 participants (China)"

***Response: We appreciate this feedback from the editor. We first revised the title of Table 1-1 from “CLHLS 2011” to “CLHLS 2012”, because data for the overall 2011-2012 wave was collected in 2012. Moreover, we rephrased the Table 2 title and all other table titles that have the same problem as below:***

***Table 2. Odds ratio (95% CI) of factors associated with CKD in Chinese participants (CLHLS 2012).***

***Table 3. Odds ratio (95% CI) of factors associated with CKD in US participants (NHANES 2011-2014).***

***Table 4. Hazard ratio (95% CI) of biomarkers on mortality in Chinese participants (CLHLS 2012).***

***Table 5. Hazard ratio (95% CI) of biomarkers on mortality in US participants (NHANES 2011-2014).***

10. In the Supplementary Table 1. Cohorts’ characteristics of this study it is indicated "Older men and women aged 65 or above" for both cohorts. However, the NHANES includes also persons 18-64 years old. Please could you explain?

***Response: We thank the editor for this question. The original NHANES data covered all-age US population. In this study, we excluded those aged below 65 years old, and only looked at the elderly aged 65 or above. We revised the description of study participants in the Supplementary Table 1 as “Older men and women aged 65 or above were included in this study” to make it clear.***

Reviewer #1: The authors' changes have improved the manuscript a great deal. Some light copy-editing is needed. One last change I would recommend is including the limitation of much higher loss to follow-up in the CLHLS sample versus the NHANES sample.

***Response: We thank the reviewer for this feedback. We added this point at the end of the Discussion (Line 621-625):***

***“Last but not least, there were more participants lost to follow-up in CLHLS than in NHANES. Those lost to follow-up in CLHLS had similar characteristics with those did not lost to follow-up except that they were more likely to have formal education and higher household income. This can make the mortality analyses results of two countries less comparable to a small extent.”***

Reviewer #2: As a consequence of correcting the text to the recommendations, the article has greatly improved.
Some minor elements persist, some already mentioned previously.
1. In the Methods chapter, on page 9 when describing "Measurement of biomarkers. In CLHLS, the urine was tested for microalbumin" should refer to albumin and not to microalbumin.
2. The diagnosis of hypertension should include, in addition to the presence of BP <140/90, the intake of antihypertensive medication
3. When describing the prevalence of CKD in both populations, some findings are contrary to the usual occurrence, so must be made some comment. In Chinese participants, those who never smoked and did physical activity, a higher prevalence of CKD was found, this finding should be commented, which is surely more in relation to age than to these characteristics, and is contrary to the effects of smoking and physical activity in general.

***Response: We thank the reviewer for giving these helpful comments. We have revised them accordingly.***

***First, we double-checked the definition of microalbumin and albumin, and then changed the item “microalbumin” into “albumin”.***

***Second, we presumed that the comparability of hypertension-related results is more important than the accuracy of hypertension definition in this comparative study. Since there was no medication data in CLHLS, we used the current definition of hypertension so that it was applicable for all participants in both countries.***

***Third, we explained this counterintuitive result in Line 538-543:***

***“Furthermore, contrary to the positive health effect of physical activity in general, we found no physical activity was associated to lower risk of having CKD among Chinese participants. We speculated that the presence of CKD might be more related to age than to physical activity. It is possible that doing exercise enable people to live longer but, thus, have CKD. This may also explain why never smokers among Chinese elderly showed a higher prevalence of CKD.”***
