# Supplementary material for: Chronic kidney disease biomarkers and mortality among older adults: A comparison study of survey samples in China and the United States
Source: PLoS One. 2022 Jan 12;17(1):e0260074. doi: 10.1371/journal.pone.0260074 (PMC8754291; doi:10.1371/journal.pone.0260074)
Supplement: Attachment — Submitted filename: rebuttal letter final.docx [file pone.0260074.s012.docx]

***Dear Editor,***

***Thank you for the opportunity for an additional round of revision on our manuscript. Our point-by-point response are below.***

Reviewer #1: This manuscript has improved, but a few remaining issues need to be resolved.

1. In the Abstract conclusion, you state that "the association between CKD and mortality was also stronger among the US older adults. This may be due to age difference, biological differences, or co-morbid conditions." However, the statistical models control for age, and so the estimated hazard ratios control for age. So age differences would then not contribute to the difference in strength of association. I suggest removing "age difference" from the last sentence.

***Response: We thank the reviewer for pointing this out. We removed “age difference” from the last sentence of the abstract (Line 53-54):***

***“This may be due to the biological differences, or co-morbid conditions.”***

2. The last sentence of the Introduction seems states a result rather than a hypothesis. Restate as a hypothesis.

***Response: We thank the reviewer for this feedback. We revised the sentence as below (Line 101-103):***

***“In addition, the association between CKD and mortality might also be different among the US elderly population and the Chinese.”***

3. Check all references for accuracy (line 94 says "refs", for example, rather than reference numbers).

***Response: We thank the reviewer for the reminding. We found Line 94-95 did not need a reference, since it referred to the articles mentioned above. Therefore, we removed “(refs)”, and revised the sentence as below. We also checked all references carefully.***

***“However, these previous studies mainly focused on comparing the prevalence of CKD in the general population between China and the US, the differences in the prevalence of CKD among elderly populations between China and the US, and their association with mortality was not known yet.”***

4. Line 243 states that "age was adjusted for as categorical in the logistic models" but does not state how. Better would be "age was included as four separate age bins (65-69, 70-74, 75-79, 80+) in the logistic models".

***Response: We appreciate the reviewer for this helpful comment. We have rephrased as below in Line 248-249:***

***“Besides, age was adjusted for as four separate age bins (65-69, 70-74, 75-79, 80+) in the logistic models and as continuous in the Cox models.”***

5. Line 518-526 discusses the rationale for excluding sample weights from the current analysis. I find this reasoning unconvincing, especially because CLHLS oversampled rural areas and NHANES minorities and lower SES, and the non-weighted results are therefore clearly non-representative. The analysis including sample weights should be in the main text, especially as NHANES has not issued guidance to ignore sample weights in that survey.

***Response: We concur with the reviewer that weighting the sample would allow us to make generalizable statements about the population at large. However, we prefer not to include the weights in the main text for reasons stated in the last revision, plus we are not making population at large statements. Treating each participant as an individual allows us to avoid some bias that may be introduced by weights, and since the motivation of our study is to look at epidemiological dose-response relationship, rather make population prevalence estimates, our method is perhaps more robust statistically. Furthermore, we presented both weighted and unweighted results for the reader. Once again, we concur with the reviewer, but prefer to exercise our discretion as authors in making this decision. If necessary, we hope the editor can make a recommendation as well.***

Reviewer #2: The questions posed have been answered in an acceptable way. In its new edition the article has been vastly improved, and is fit to be published.

***Response: We the reviewer for the feedback.***
