# Supplementary material for: Chronic kidney disease biomarkers and mortality among older adults: A comparison study of survey samples in China and the United States
Source: PLoS One. 2022 Jan 12;17(1):e0260074. doi: 10.1371/journal.pone.0260074 (PMC8754291; doi:10.1371/journal.pone.0260074)
Supplement: Attachment — Submitted filename: rebuttal letter.docx [file pone.0260074.s013.docx]

***Dear Editor,***

***Thank you for the opportunity for an additional round of revision on our manuscript. The reviewer was very fast in providing additional feedback, we appreciate this. To address the final edit, our point-by-point response are below.***

Reviewer #1: I thank the authors for their quick response. I accept that they will not include sample weights in the main analysis of the paper and am glad the Supplement includes them, and I now suggest two remaining changes:

1. Change statements that assert national representativeness

In the authors' last response, they state "However, we prefer not to include the weights in the main text for reasons stated in the last revision, plus we are not making population at large statements. Treating each participant as an individual allows us to avoid some bias that may be introduced by weights, and since the motivation of our study is to look at epidemiological dose-response relationship, rather make population prevalence estimates, our method is perhaps more robust statistically." In the Results section, the authors are careful to restrict statements to the study samples, but there remain many sentences that do still refer to "the US" or "China" (or similar generalized references), and there are many sentences that do make population prevalence estimates, contrary to the authors' response. Before publication, results need to be changed to refer to the study samples (such as "CLHLS sample" or "US participants", etc) rather than the countries in all of the following lines:
282-285 (example wording change included below), 287, 303, 305, 309, 311, 312, 313, 315-326 ("both countries" change to "both samples"), 332 ("two countries" change to "both samples"), 343, 362, 421, 423, 442, 450, 456.

Original text for lines 282-285: "Among people over 65 years of age, the prevalence of CKD in China and the US were 44.4% (95% CI: 42.2%-46.6%) and 42.3% (95% CI: 40.2%-44.6%), respectively. Besides, the CKD prevalence for the participants without diabetes and hypertension were 37.8% in China and 33% in the US (diabetes/hypertension prevalence: 58% in China vs. 50% in the US)."
Suggested change: "Among people over 65 years of age, the prevalence of CKD in the CLHLS and the NHANES samples were 44.4% (95% CI: 42.2%-46.6%) and 42.3% (95% CI: 40.2%-44.6%), respectively. Besides, the CKD prevalence for the participants without diabetes and hypertension were 37.8% in CLHLS and 33% in NHANES (diabetes/hypertension prevalence: 58% in CLHLS vs. 50% in NHANES)."

***Response: We concur, and revised the wording as below:***

***Title has been revised to:***

***“Title: Chronic Kidney Disease Biomarkers and Mortality among Older Adults: A Comparison Study of Survey Samples in China and the United States”***

***Line 282-286:***

***“Among people over 65 years of age, the prevalence of CKD in CLHLS and NHANES samples were 44.4% (95% CI: 42.2%-46.6%) and 42.3% (95% CI: 40.2%-44.6%), respectively. Besides, the CKD prevalence for the participants without diabetes and hypertension were 37.8% in CLHLS and 33% in NHANES (diabetes/hypertension prevalence: 58% in CLHLS vs. 50% in NHANES).”***

***Line 286-287:***

***“However, the prevalence in each age group of NHANES participants was higher than Chinese participants, …”***

***Line 304:***

***“… were lower in the Chinese participants than the US sample …”***

***Line 306:***

***“… while the BUN (mmol/L) was higher in the Chinese sample (6.9 vs 6.1) (Table 1).”***

***Line 309-315:***

***“The mean difference between the CLHLS and NHANES varied in different age groups. The urinary creatinine, BUN and eGFR were both higher in CLHLS participants than NHANES sample aged younger than 80 and this difference almost disappeared in participants aged 80 and older. The eGFR decreased with age increasing in both CLHLS and NHANES samples. The urinary albumin, ACR, serum creatinine, and uric acid were lower in Chinese participants than the US ones among different age groups consistently. Plasma albumin level was similar in both samples across all age groups.”***

***Line 317-328:***

***“There was a large difference in the urinary albumin between the two samples. The mean of urinary albumin level of CLHLS participants was 25.0 mg/L (SD: 75.4), much lower than that of NHANES participants (76.4 mg/L, SD: 359.6). Among Chinese participants, higher urinary albumin level usually appeared along with factors like age over 80 (29.5 mg/L), female (25.8 mg/L), high household income (29.8 mg/L), widowed (28.6 mg/L), good health condition (25.7 mg/L), never smoker (26.6 mg/L), and former drinker (34.7 mg/L) (Table 1-1). However, among NHANES participants, people had higher urinary albumin were more likely to be aged 75-79 (109.3 mg/L), male (98.1 mg/L), with low household income (88.8 mg/L), separated (134.2 mg/L), with poor health condition (171.8 mg/L), current smoker (111.6 mg/L), and never drinker (98.0 mg/L) (Table 1-2). Besides, some characteristics were found related to higher urinary albumin level in both samples, including lower education, obese, insufficient physical activity, hypertension, and diabetes (Table 1-1 & 1-2).”***

***Line 332-334:***

***“However, we did not find a similar pattern in the two samples in terms of the relationship between ACR and other demographic or lifestyle characteristics.”***

***Line 345:***

***“There were different factors associated with CKD between CLHLS and NHANES samples.”***

***Line 363-365:***

***“Among NHANES participants, current smokers also showed about 40% greater odds of having CKD. Besides, in the Chinese sample, those who had no physical activities had lower odds of CKD (0.708, 95% CI: 0.534, 0.938) than the others.”***

***Line 424-427:***

***“In the US sample, gender was not associated with CKD, while high household income and low education level were associated with a higher prevalence of CKD. Furthermore, the association between CKD/eGFR and mortality was found stronger in the US sample.”***

***Line 445:***

***“Although the mean eGFR in our study was lower in in the Chinese sample, …”***

***Line 452-453:***

***“This study also suggested that being female was associated with decreased eGFR in CLHLS participants but not in the NHANES sample, …”***

***Line 458-460:***

***“The weaker relationship between higher BMI and CKD may be reasonable considering the huge gap between the proportion of overweight people in the Chinese and the US samples.”***

2. Clarify that the sample weight limitation applies to both surveys.

Lines 525-526 original text: "We only presented the results considering sample weight as supplementary materials."

Suggested change: "We excluded sample weights from CLHLS for this reason and from NHANES for consistency, and we present results incorporating the sample weights for both studies in the supplementary materials."

I thank the authors for the opportunity to provide feedback on this manuscript, and I look forward to seeing it published soon.

***Response: We revised the sentence as suggested (Line 529-531):***

***“We excluded sample weights from CLHLS for this reason and from NHANES for consistency, and we present results incorporating the sample weights for both studies in the supplementary materials.”***
